# Novel frontiers in urogenital cancers: from molecular bases to preclinical models to tailor personalized treatments in ovarian and prostate cancer patients

**DOI:** 10.1186/s13046-024-03065-0

**Published:** 2024-05-15

**Authors:** Giada De Lazzari, Alena Opattova, Sabrina Arena

**Affiliations:** 1 Candiolo Cancer Institute, FPO - IRCCS, Laboratory of Translational Cancer Genetics, Strada Provinciale 142, Km 3.95, Candiolo, TO ZIP 10060 Italy; 2 Department of Oncology, University of Torino, Strada Provinciale 142, Km 3.95, Candiolo, TO ZIP 10060 Italy

**Keywords:** Genomic instability, Targeted-therapies, Chemotherapies, DNA damage response, Ovarian cancer, Prostate cancer, Hormonal regulation, Androgen receptor, Preclinical models, Organoids

## Abstract

Over the last few decades, the incidence of urogenital cancers has exhibited diverse trends influenced by screening programs and geographical variations. Among women, there has been a consistent or even increased occurrence of endometrial and ovarian cancers; conversely, prostate cancer remains one of the most diagnosed malignancies, with a rise in reported cases, partly due to enhanced and improved screening efforts.

Simultaneously, the landscape of cancer therapeutics has undergone a remarkable evolution, encompassing the introduction of targeted therapies and significant advancements in traditional chemotherapy. Modern targeted treatments aim to selectively address the molecular aberrations driving cancer, minimizing adverse effects on normal cells. However, traditional chemotherapy retains its crucial role, offering a broad-spectrum approach that, despite its wider range of side effects, remains indispensable in the treatment of various cancers, often working synergistically with targeted therapies to enhance overall efficacy.

For urogenital cancers, especially ovarian and prostate cancers, DNA damage response inhibitors, such as PARP inhibitors, have emerged as promising therapeutic avenues. In *BRCA*-mutated ovarian cancer, PARP inhibitors like olaparib and niraparib have demonstrated efficacy, leading to their approval for specific indications. Similarly, patients with DNA damage response mutations have shown sensitivity to these agents in prostate cancer, heralding a new frontier in disease management. Furthermore, the progression of ovarian and prostate cancer is intricately linked to hormonal regulation. Ovarian cancer development has also been associated with prolonged exposure to estrogen, while testosterone and its metabolite dihydrotestosterone, can fuel the growth of prostate cancer cells. Thus, understanding the interplay between hormones, DNA damage and repair mechanisms can hold promise for exploring novel targeted therapies for ovarian and prostate tumors.

In addition, it is of primary importance the use of preclinical models that mirror as close as possible the biological and genetic features of patients’ tumors in order to effectively translate novel therapeutic findings “from the bench to the bedside”.

In summary, the complex landscape of urogenital cancers underscores the need for innovative approaches. Targeted therapy tailored to DNA repair mechanisms and hormone regulation might offer promising avenues for improving the management and outcomes for patients affected by ovarian and prostate cancers.

## Background

In this review, we aimed to explore the complex landscape of urogenital cancers, with a specific focus on the current therapeutic approaches available, particularly for ovarian and prostate cancer. We highlight the pivotal roles played by genomic instability and DNA repair mechanisms in both the development and treatment of these malignancies. We emphasize the crucial impact of mutations in DNA repair genes, which have paved the way for targeted therapeutic interventions. Furthermore, we underscore the intricate interplay between hormonal dysregulation and DNA damage, suggesting potential for new treatment modalities. Finally, we shed light on the importance of advanced models as genetically engineered mouse models, patient-derived xenografts and organoids. These models not only mimic human cancers more accurately, but also serve as indispensable tools in guiding the development of tailored therapies in the frame of a precision medicine approach in the battle against urogenital cancers.

### Urogenital cancers: insights from ovarian and prostate tumorigenesis

Urogenital cancers encompass a diverse array of malignancies affecting the urinary and reproductive systems, arising from organs like the kidneys, bladder, prostate, testicles, ovaries, uterus, and related structures [1]. Each cancer type within this spectrum possesses unique characteristics, risk factors, and treatment approaches [2]. Therefore, early detection, accurate diagnosis, and timely intervention are crucial for improving outcomes in individuals diagnosed with these cancers [1]. A fundamental aspect of cancer development lies in the role of DNA repair mechanisms. In healthy cells, DNA repair mechanisms accurately fix genetic damage, preserving genomic stability. However, compromised repair systems lead to the accumulation of DNA damage, resulting in accumulation of mutations and genomic instability, which are key hallmarks of cancer [3, 4]. Inherited defects in DNA repair genes, such as *BRCA1/2* in breast, ovarian and prostate cancers, significantly increase the risk of tumor development [3]. Tumors exploiting deficient repair pathways become reliant on alternative mechanisms, driving genomic instability and cancer progression. This understanding of repair deficiency in cancer cells has led to the identification of specific therapeutic targets [5]. For instance, as demonstrated by González-Martín and colleagues, cancers with impaired homologous recombination (HR) are particularly sensitive to PARP inhibitors (PARPi) and the authors demonstrate the effectiveness of niraparib as specific therapeutic agent against HR in treating patients with ovarian cancer [6]. Given the critical role of DNA repair pathways in cancer progression, in this review we will delve these mechanisms focusing on two main subtypes of urogenital cancer, ovarian and prostate tumors.

Both these cancers, while distinct in their manifestation and impact on different genders, share common ground in the molecular dysregulation of cellular processes, including DNA repair pathways and common mutation in genes such as *BRCA1/2* [7, 8].

Ovarian cancer (OC), often termed as the “silent killer,” is the sixth most common cancer and the fifth for mortality in women and it poses unique challenges due to its asymptomatic nature in early stages [2, 9]. Globally, the incidence and mortality rates of OC exhibit considerable geographical variability: higher incidence is shown in Northern Europe and the United States and lower in Japan while its mortality has exhibited a notable decrease from 2017 through 2020 [2, 10]. The etiology of OC is multifaceted, implicating a range of risk factors. Advanced age emerges as a significant contributor, with the majority of cases diagnosed in postmenopausal women [9, 11]. The pathophysiology of OC involves the dysregulation of key cellular processes, including uncontrolled cell proliferation and evasion of apoptosis, often leading to the formation of epithelial tumors [12, 13]. Diagnostic strategies for OC encompass protein and imaging diagnostics, along with preoperative assessments, employing methods like different index assays as described in the work of Liberto and colleagues [14]. As pointed out in this and other works [14–16] a panel of four marker for OC diagnosis including CA125, CA72-4, CA15-3, and MCSF can help in increasing the sensitivity of the technology. Together with protein markers also imaging diagnostics have evolved; imaging techniques, such as ultrasound and magnetic resonance imaging (MRI), help in visualizing tumors and assessing their extent [17, 18]. The complexity of OC is also reflected on treatment modalities since surgical interventions, including hysterectomy and oophorectomy are often employed as first line treatment [19]. The surgical approach is often reinforced by chemotherapy, with agents like cisplatin, carboplatin and taxanes (e.g. paclitaxel) and targeted therapies such as PARPi in specific genetically altered tumors [19, 20]. Preventive strategies and screening programs are integral components of the comprehensive approach to urogenital cancers. Risk-reducing measures, such as prophylactic surgery for individuals with high-risk genetic mutations, offer a preventive option for OC [21, 22]. However, challenges persist in developing effective screening methods for OC due to its often asymptomatic nature in early stages [21, 22]. Moreover, OC distinctly highlights how genetic and molecular dysregulations in the urogenital tract can lead to malignancy. Genetic mutations, notably in the *BRCA1* and *BRCA2* genes but also in *TP53*, *KRAS* and *PIK3CA*, are central to understand this type of tumor since they highlight broader tumorigenic processes across OC [23, 24]. In fact, beyond their known role in double-strand DNA break repair pathways and in particular in the regulation of HR, these mutations also have other functions such as being a regulator of oxidative stress and cell cycle progression (*BRCA1*) or being involved in transcriptional regulation (*BRCA1/2*) [25, 26]. In this context, starting from the main function of these genes, researchers have increasingly emphasized the analysis of the link between their dysregulation and tumorigenesis and consequently the study of homologous recombination repair (HRR) deficiencies which has led to significant therapeutic advancements on urogenital cancers [26–28]. Moreover, the observed heterogeneity in ovarian tumor cells, including variations in the tumor microenvironment and metabolic pathways, offers a deeper understanding of tumorigenesis. The intricate interactions within the ovarian tumor microenvironment, involving stromal cells, immune evasion mechanisms, and angiogenesis, further elucidate the complexities of tumorigenesis in the urogenital system. This understanding is pivotal in developing targeted therapeutic strategies, as it reveals how cancer cells manipulate their surroundings for survival and growth. Moreover, the metabolic adaptations seen in OC cells provide insights into potential vulnerabilities that could be therapeutically exploited, indicating how metabolic dysregulation in the urogenital tract can contribute to cancer development [29].

Prostate cancer (PC) is the most common type of solid cancer and the second cause of cancer-related death in men [2]. The etiology of PC includes different types of risk factors such as age, race, family history, and germline mutations (*BRCA1/2, CHEK2, ATM*) [30]; in addition, metabolic syndrome, obesity, and smoking have been identified as possible risk factors [31]. PC is characterized by different stages, from intraepithelial neoplasia and localized PC, to the advanced prostate adenocarcinoma with local invasion. The most advanced stage, metastatic PC (mPC), is characterized by the invasion of other different organs and tissues in the body. For the grading of PC, the Gleason grading system is used [32]. Early detection is crucial for successfully treating PC. Various screening methods aim to improve cancer detection in its early stages, with the prostate-specific antigen (PSA) test being the most widely promoted and FDA-approved method since 1986. PSA, typically found at low levels in the blood, becomes elevated in the presence of prostatic disease due to disruption in organ microarchitecture [33]. However, the low specificity of the PSA test necessitates additional measures to reduce unnecessary prostate biopsies, leading to the development of the prostate health index (PHI) blood test. This test combines free and total PSA with the (− 2) pro-PSA isoform (p2PSA) to enhance accuracy [34]. Recent studies showed Prostate Cancer Antigen 3 (PCA3) as overexpressed in 95% of PC cases, leading to the development of a non-invasive urine PCA3 test for screening [35]. Usually, the screening starts for 50-year-old men, but for high-risk individuals (germline mutations in *BRCA1, BRCA2, ATM, CHEK2*; family history of PC) the screening should commence as early as age 40 [36]. Diagnostic strategies for PC include MRI combined with dynamic contrast-enhanced MRI and more specific Prostate-Specific Membrane Antigen (PSMA) positron emission tomography PET/CT [37].

PC is well known by high morphological and genetic heterogeneity [38]. The main genetic alterations in PC affect androgen receptor (AR), Phosphatidylinositol-3-kinase/ Phosphatase and tensin homolog *(PIK3CA–PTEN*), *WNT*, and genes involved in DNA repair signaling pathways (*BRCA1, BRCA2, ATM, CHEK2*) [39].Treatment options for PC depend on the stage of the disease. For localized disease, active surveillance, radical prostatectomy, or ablative radiotherapy are employed. Patients with localized disease show a favourable outcome if the disease is early detected and treated. For the advanced stages, radiotherapy and/or androgen deprivation therapy are used. For the mPC, AR-targeted agents, chemotherapy (taxanes), and radionuclides are used [40]. As we already mentioned, PC is characterized by the presence of DNA repair mutations, which increases in the metastatic setting of the PC. Therefore, the PARPi olaparib has been approved for use in patients with *BRCA2* mutations [41]. However, after an initial response, PC can progress in developing castration resistance (CRPC), posing ongoing challenges in disease management.

In this first section of this review, we aimed to explore urogenital cancers tumorigenesis which helps our understanding of these particular type of cancers but also provides critical insights into the mechanisms of cancer development. Both tumors share notable similarities for example in DNA damage and repair mechanisms, hormonal regulation and key tumor characteristics. They both frequently exhibit defects in DNA damage repair (DDR) pathways, such as homologous recombination (common mutations in *BRCA* genes) [42], and hormone regulation plays a significant role in both, with estrogen receptor signaling influencing OC and androgen receptor pathways being pivotal in PC [43]. Additionally, both cancers often develop resistance to hormone-based therapies and may respond to PARPi, highlighting shared therapeutic vulnerabilities [44]. Due to these similarities, from now on this review will be mainly focused on mechanisms of DNA damage and repair and hormonal regulation in the context of OC and PC by evaluating the currently available therapeutic strategies and preclinical available models for both cancers.

### Deconvolution of urogenital cancer complexity

Exploring the role of OC and PC in the urogenital tract tumorigenesis lays the groundwork for understanding cancer’s broader complexities. This exploration extends to the fundamental framework of the hallmarks of cancer, delving into genetic instability and synthetic lethality, which are pivotal in comprehending the multifaceted nature of cancer. In this scenario, cancer research underwent a paradigm shift with the introduction of the “Hallmarks of Cancer” by Hanahan and Weinberg in their 2000 publication [45]. This concept delineates a set of mechanisms acquired by human cells during their transition from normal to neoplastic states, crucial for malignant tumor development [45]. Initially, Hanahan and Weinberg outlined six biological capabilities acquired during the multistep development of human tumors, such as insensitivity to antigrowth signals, evasion of apoptosis, sustained angiogenesis, limitless replicative potential, tissue invasion and self-sufficiency in growth signals [46]. Subsequently, this list was expanded to eight hallmarks and two enabling characteristics by incorporating tumor-promoting inflammation, genome instability and mutation and the ability of cancer cells to often undergo changes in their metabolism and to avoid immune system destruction [47]. Among these hallmarks, “Genome Instability and Mutation” holds a central position, driving the acquisition of other hallmarks.

Genomic instability, defined as an increased susceptibility of a cell's genome to acquire mutations, stems from defects in DNA repair mechanisms, replication errors, exposure to mutagenic agents, or other genetic or environmental factors, leading to high mutation rate and resulting in a heterogeneous tumor population with diverse genetic compositions [48, 49].

In the context of OC, one of the most significant implications of genomic instability is the development of resistance both primary and secondary to platinum-based chemotherapy, a cornerstone of its treatment [50–52]. Primary resistance occurs when cancer cells exhibit intrinsic resistance to therapeutic agents, while secondary (acquired) resistance develops over time, likely due to adaptation to treatment selection pressure [50, 53] For instance, alterations in the *BRCA1/2* genes, which are crucial for HRR, are common in OC and can confer initial sensitivity to platinum-based therapies. During treatment, the occurrence of reversion mutations in these genes can restore lost repair function, leading to drug resistance [51, 54]. Furthermore, other recent studies have identified additional genetic alterations that contribute to platinum resistance in OC, such as mutations in *RAD51C* and *RAD51D*, which further complicate the treatment landscape [55, 56]. Additionally, the high degree of genomic instability in OC can correlate with tumor heterogeneity, as demonstrated by Bashashati and colleagues [57], revealing distinct genetic profiles among tumor subclones that may respond differently to the therapy In line with this, researchers start to explored the implications of intratumor heterogeneity in OC prognosis, emphasizing the need for personalized treatment approaches [58]. Liquid biopsy technologies offer dynamic and precise monitoring of these genetic variations, aiding in the assessment of treatment response and disease progression [58–60]. The genomic instability of both OC and PC has also opened new avenues for targeted therapy. PARPi, for example, exploit the concept of synthetic lethality in cancer cells deficient in HRR as seen in *BRCA*-mutated OC and PC [61, 62]. Recent advancements in this area have shown promising results in the use of PARPi in prolonging progression-free survival especially in patients carrying *BRCA* mutation and HRD-positive status [63, 64]. However, the adaptive capacity of cancer cells due to genomic instability presents an ongoing challenge. This adaptive nature of cancer due to its genomic instability, not only leads to challenges like chemoresistance and tumor heterogeneity, but also paves the way for innovative therapeutic strategies, such as those exploiting synthetic lethality [65]. In a synthetically lethal relationship, the simultaneous impairment of two genes or pathways leads to cell death, whereas the disruption of either alone is tolerable to the cell. This concept is particularly relevant in cancer cells, which often harbour specific genetic mutations making them susceptible to targeted therapies that exploit their inherent genetic weaknesses [65]. PC and OC are a prime candidate for therapies based on synthetic lethality; indeed, *BRCA* mutations impair the HR DNA repair pathway, making the cancer cells more dependent on alternative repair mechanisms [66]. This dependency creates an opportunity for targeted therapy as we have already discussed. More recent studies have expanded on these findings exploring the broader implications of synthetic lethality focusing especially on the combination therapies that integrate synthetic lethality concepts. Lord and Ashworth investigated the synergistic effects of combining PARPi with other targeted agents, offering novel strategies to overcome resistance mechanisms that OC cells develop in response to monotherapy [5]. While genomic instability poses significant challenges in the form of chemoresistance and tumor heterogeneity, it also provides opportunities for developing innovative targeted therapy strategies. The latest studies in the field reinforce the potential of synthetic lethality in offering effective, personalized treatment options for OC, catering to its adaptive nature and genetic diversity.

### DNA damage: DNA repair mechanisms in ovarian and prostate tumors

As highlighted in the previous section, the ongoing research in genomic instability and synthetic lethality in OC and PC treatment sparks discussion about the intricate interplay among genomic instability, DNA damage, and repair mechanisms. Understanding these mechanisms is pivotal in this context where DDR plays a significant role in disease development and progression. A wealth of literature has been published on this topic and here we aim to provide a concise overview of the key concepts, primarily focusing on urogenital tumors.

DNA damage can be broadly categorized into two groups: single-strand breaks (SSBs) and double-strand breaks (DSBs). SSBs are the most common and are generally less harmful as the complementary DNA strand remains intact, serving as a template for repair. In contrast, DSBs are more critical and can lead to significant genomic instability if not appropriately repaired [47]. This distinction is crucial in the context of urogenital cancers, where genetic material integrity is paramount for cell function [67].

Cells have evolved several mechanisms to repair damaged DNA, each tailored to specific types of damage. These includes Nucleotide Excision Repair (NER), which is primarily responsible for repairing bulky DNA lesions caused by UV radiation and certain chemicals; Base Excision Repair (BER) which corrects small, non-helix-distorting base lesions caused by oxidation or methylation. In addition, Mismatch Repair (MMR) corrects errors that occur during DNA replication. Defects in MMR are known to contribute to the development of certain types of cancers, including urogenital cancers [47]. Finally, HR and Non-Homologous End Joining (NHEJ) are two critical pathways for repairing DSBs. HR is an error-free repair process utilizing a sister chromatid as a template for repair, while NHEJ is an error-prone process directly joining broken end [49]. These cancers often exhibit inherent defects in DNA repair pathways, particularly in HR [68]. DNA damage and repair mechanisms are critically linked to the therapeutic potential of DDR inhibitors (DDRi). These inhibitors, such as PARPi, target mechanisms that cancer cells rely on for survival and proliferation exploiting the concept of synthetic lethality [46]. As discussed in the previous paragraph, *BRCA1* and *BRCA2* mutations impair HR repair in OC and make OC cells particularly vulnerable to PARPi. By inhibiting PARP enzymes, which play a crucial role in single-strand break repair, these drugs exacerbate DNA damage in cells already compromised in their ability to repair double-strand breaks, leading to cell death. For this reason, this approach is often use therapeutically [69] and clinical trials with PARPi in OC are extensively reported in different studies [70–72]. However, the scope of DDRi extends beyond PARPi and *BRCA* mutations. Recent studies have shown that other DDR pathways and inhibitors are also clinically significant; indeed, we will focus on other DDRi such as ATRi, CHK1i, WEE1i and DNA-PKi. For instance, inhibitors targeting the ATR-CHK1-WEE1 axis, which are key components of the DDR involved in the cell cycle checkpoint regulation, have shown promise in preclinical models of OC [73]. These inhibitors can enhance the effects of DNA-damaging chemotherapy and radiation therapy, offering a potential combinatorial approach to cancer treatment. For this reason, several clinical trials based on ATR-CHK1-WEE1 axis are further exploring this avenue (Table 1) [47]. Addressing this challenge requires a deeper understanding of resistance mechanisms and the development of next-generation DDR inhibitors able to overcome it [74]. When developing new DDRi, tailoring treatments based on individual genetic profiles is imperative. As suggested in the work of Foster and colleagues, genomic sequencing can identify specific DNA repair deficiencies in tumors, guiding the selection of appropriate DDR inhibitors [75]. This precision medicine approach ensures that patients receive the most effective treatment tailored to their unique cancer biology.

**Table 1:**
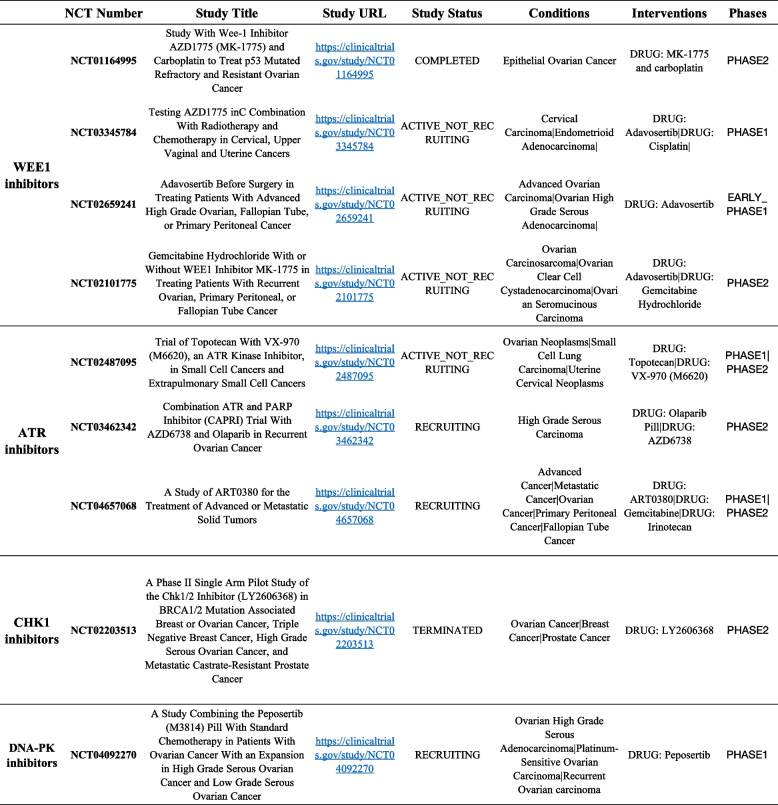
Ovarian cancer clinical trials with DDR inhibitors. This table summarizes the main clinical trials testing WEE1i, ATRi, CHK1i and DNA-PKi and available on the website https://clinicaltrials.gov/

Comprehensive molecular characterization of PC has revealed a significant inter-patient genomic heterogeneity and phenotypic diversity. The most prominently altered pathways include androgen signaling (50%), PI3K signaling (40%), the cell cycle (24%), WNT/beta-catenin signaling (19%), RAS pathway (8%) [76, 77] along with DDR pathways (27%) [78]. Recent studies have indicated that germline mutations in DDR genes are associated with a higher risk of developing PC and worse clinical outcomes as well as with aggressive phenotype with increased probability to develop metastasis [79]. Approximately 10–19% of primary PCs exhibit somatic alterations in DDR genes, with this number increasing to 23–27% in the metastatic setting. Mateo and colleagues showed differences in *AR, TP53, RB1*, and *PI3K/AKT* mutational status between matched hormone-naive and metastatic castration-resistant prostate cancer (mCRPC) biopsies [80]. Furthermore, multicentric study on a cohort of 150 mCRPC showed increased aberrations of *BRCA2, BRCA1* and *ATM* (19.3%) compared to primary PCs [81]. Taken together this introduces important prognostic value of DDR mutations. Current studies and clinical trials indicated that alterations in DDR genes also contribute to disease progression and therapy response in PC [41]. Initially identified mutations in DDR genes were *BRCA1* and *BRCA2* genes, followed by discoveries of germline or somatic mutations also in other DDR genes e.g.: *ATM, CDK12, FANCA, RAD51B,* and *RAD51C, CHEK2* in PC [76, 82]*.* Inactivating mutations in these tumor suppressor genes increase predisposition to PC. Moreover, loss-of-function mutations of DDR-associated genes leads to a deficiency in error-free HR repair. DSBs are then repaired by alternative repair pathways that are more error-prone, e.g. NHEJ. Consequently, these lead to the genetic instability of the tumor. Despite this, these genes present potential therapeutic targets in PC [41]. Increasing evidence suggests that other DNA repair pathways, such as a MMR and BER, may play an important role in PC. Approximately 4% of PC tumors and 6% of metastatic PCs (mPC) had alterations in *MSH2* and *MSH6*, with clinical implications such as resistance to immune checkpoint inhibitors (ICIs) noted in MMR-deficient patients [83]. Vasquez and colleagues showed that upregulation of BER related genes is associated with poor survival in PC patients, with inhibition of BER by natamycin significantly impaired PC cells proliferation in androgen depleted PC [84]. As pointed out before, genome instability is one of the important hallmarks of cancer [4] and DDR is responsible for the maintenance of genome integrity. In PC, cancer cells frequently harbour DDR gene deficiencies, providing a potential avenue for targeting DDR to induce cancer cell death. The PARPi olaparib was initially approved for the treatment of advanced ovarian and breast cancers associated with germline *BRCA1* or *BRCA2* mutations [85]. Clinical trials such as the TOPARP have demonstrated high response rates to PARPi in patients with DDR gene defects [39]. The clinical trial TOPARP-B studied the antitumour activity of olaparib against mCRPC with DDR gene aberrations [86]. Similar results have been obtained in clinical trials with rucaparib [87]. Based on these studies PARPi were approved by FDA for PC treatment in 2020 and the importance of these pathways in PC therapy response is also confirmed by the number of clinical trials already performed or currently ongoing [88]. Additionally, ongoing trials focusing on components like the ATR-CHK1-WEE1 axis suggest potential novel therapeutic options, as single agents or combinations, for PC. Drapela and colleagues showed synergistic effect of CHK1 inhibitor MU380 with gemcitabine in in vitro model of CRPC [89]. ATR inhibition led to the destabilization of PD-L1 protein in vitro. This indicates potential possibility to use of ATRi in combination with immune checkpoint blockade as a novel therapy option [90]. Examples of ongoing clinical studies focused on ATR-CHK1-WEE1 are summarized in the table below (Table 2).

**Table 2:**
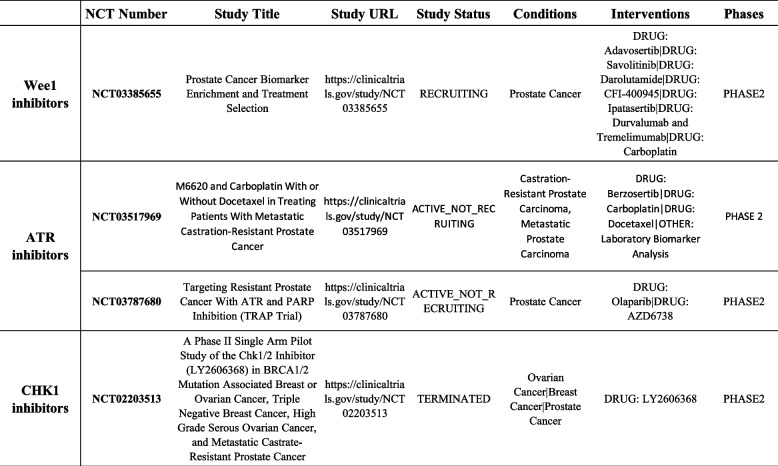
Prostate cancer clinical trials with DDR inhibitors. Clinical trials available on the website: https://clinicaltrials.gov/ are listed for each main treatment option

#### How could the combination of PARPi and immune checkpoint inhibitors (ICI) affect “cold” tumor treatment?

Immunotherapy has emerged as novel approach in the oncological landscape and among the most promising strategies in this field are immune checkpoint inhibitors (ICIs), which have revolutionized cancer treatment by promoting the body's immune system to recognize and combat tumor cells. In particular, ICIs efficacy has recently seen a relevant improvement in tumors such as ovarian ad prostate ones, that are generally considered as immunologically "cold" due to their low mutation burden and reduced immunogenicity [91, 92]. The most involved checkpoints pathways include the programmed cell death protein 1 (PD-1), PD-L1 and cytotoxic T-lymphocyte-associated protein 4 (CTLA-4) which modulate T cell function. In the PD-1/PD-L1 pathway, PD-1, a receptor expressed on T cells, binds to PD-L1, which is expressed on tumor cells and some immune cells. This interaction results in the inhibition of T cell activation and proliferation, thereby dampening the immune response against cancer cells. CTLA-4, on the other hand, reduces the activation of T cells, further downregulating the immune response [93, 94]. These immune checkpoint pathways have emerged as promising targets for cancer immunotherapy, with the development of monoclonal antibodies against PD-1 (e.g. nivolumab, pembrolizumab), PD-L1 (e.g. avelumab, atezolizumab, durvalumab) and CTLA-4 (e.g. ipilimumab) showing clinical efficacy in the treatment of various cancers [93, 95]. By inhibiting checkpoint molecules, ICIs are also beginning to show promise in overcoming the immune resistance often encountered in OC and PC treatment. Recent advancements have aimed to overcome these challenges by combining ICIs with other therapies such as chemotherapy, targeted therapy, and PARPi, which may affect the tumor microenvironment to enhance immune response [88, 96, 97]. In particular, the combination of PARPi and ICIs is being actively explored in clinical trials. In OC the main ICIs approved and used in clinical trials are pembrolizumab, nivolumab and ipilumab and they are used either alone (NCT02674061, NCT01611558 and NCT02728830) or in combination with chemotherapeutic agents such as paclitaxel (NCT03394885 and NCT02440425) and carboplatin (NCT03029598) or with PARPi such as rucaparib (NCT03824704, ARIES study) or niraparib (NCT02657889, TOPACIO study). From these clinical trials of note for their results are the TOPACIO/Keynote-162 study, the MEDIOLA study and the NCT2484404 study [98]. The TOPACIO study evaluated the combination of pembrolizumab and niraparib in recurrent platinum-resistant epithelial OC patients. The preliminary results of this study appear promising, being 4/8 evaluable OC patients responsive and the other 4 patients achieving SD, highlighting the importance of this combinatorial approach especially for OC and also other tumors with poor response to immunotherapy alone [99]. The MEDIOLA study evaluated the effect of the combination of olaparib and durvalumab (anti-PD-L1) in PARPi and ICI naïve *BRCA* mutant OC patients. As preliminary results, the combination has shown a high objective response rate (92%) in germline mutant *BRCA* patients, while the combination of olaparib, durvalumab and bevacizumab resulted as the best treatment for *BRCA* wild-type patients [100]. The results obtained from the MEDIOLA study were also confirmed by the NCT2484404 study in which the combination of olaparib and durvalumab was evaluated in patients with recurrent OC, showing also in this case a good tolerability for this treatment [101]. The encouraging results observed from this combinatorial treatment approach is fostering the design of novel clinical trial that might improve the response of OC to PD-1/PD-L1 and CTLA-4 inhibitors OC [102].

In PC, pembrolizumab has been approved only for patients with high microsatellite instability and deficient mismatch repair, which occur in 2–4% of cases [103]. There are several clinical studies to evaluate the effect of pembrolizumab alone [104, 105] and in combination with enzalutamide [106, 107] docetaxel [108] and olaparib [105] in PC. Initial data showed that only a minor subset of heavily pretreated patients can benefit from pembrolizumab therapy [104]. For example, in the Keynote 028 study, 23 patients with mCRPC positive for PD-L1 expression were enrolled and received pembrolizumab treatment, only four patients responded positively [104]. Keynote199 study showed that pembrolizumab as a monotherapy has antitumor activity in the bone-predominant mCRPC previously treated with docetaxel and targeted endocrine therapy (enzalutamide and abiraterone). This study also showed that 12% of the patients had aberrations in *BRCA1/2* or *ATM*, and 10 (7%) had alterations in 12 or more other HRR genes. None of the six patients who experienced a response with evaluable genomic data had microsatellite instability. Taken together, responders with* BRCA1/2* or *ATM* mutations had a longer response duration than responders without HRR aberrations [109]. The effect of the combination of olaparib and PD-1 has been published in several types of tumors [110]. In case of PC results of the combinational treatment with pembrolizumab and olaparib showed limited efficacy. Moreover, the efficacy was independent of HRR status and PD-L1 status [111]. When in combination, pembrolizumab plus enzalutamide in mCRPC previously treated with abiraterone showed limited antitumor activity. The phase 1b or 2 KEYNOTE-365 trial study included molecularly unselected docetaxel-treated mCRPC patients.

Recent studies indicate that ICIs alone and in combinations have only moderate effects in PC, but accurate predictive biomarkers have yet to be established for PC. Moreover, all the studies were performed on heavily pretreated and molecularly not selected patients. On a base of recent findings about pembrolizumab therapy and HRR [109], ICIs may be more effective in specific groups of molecularly selected PC patients carrying HRR defects. For example, as we have already mention above, the combination of ATR inhibition and anti-PD-L1 treatment resulted in synergistic, antitumor activity in PC [90]. This potent combination has already been tested in early-phase clinical trials in advanced malignancies (NCT04266912 and NCT04095273).

### Hormonal regulation and its implications for DNA damage and repair

**Fig. 1:**
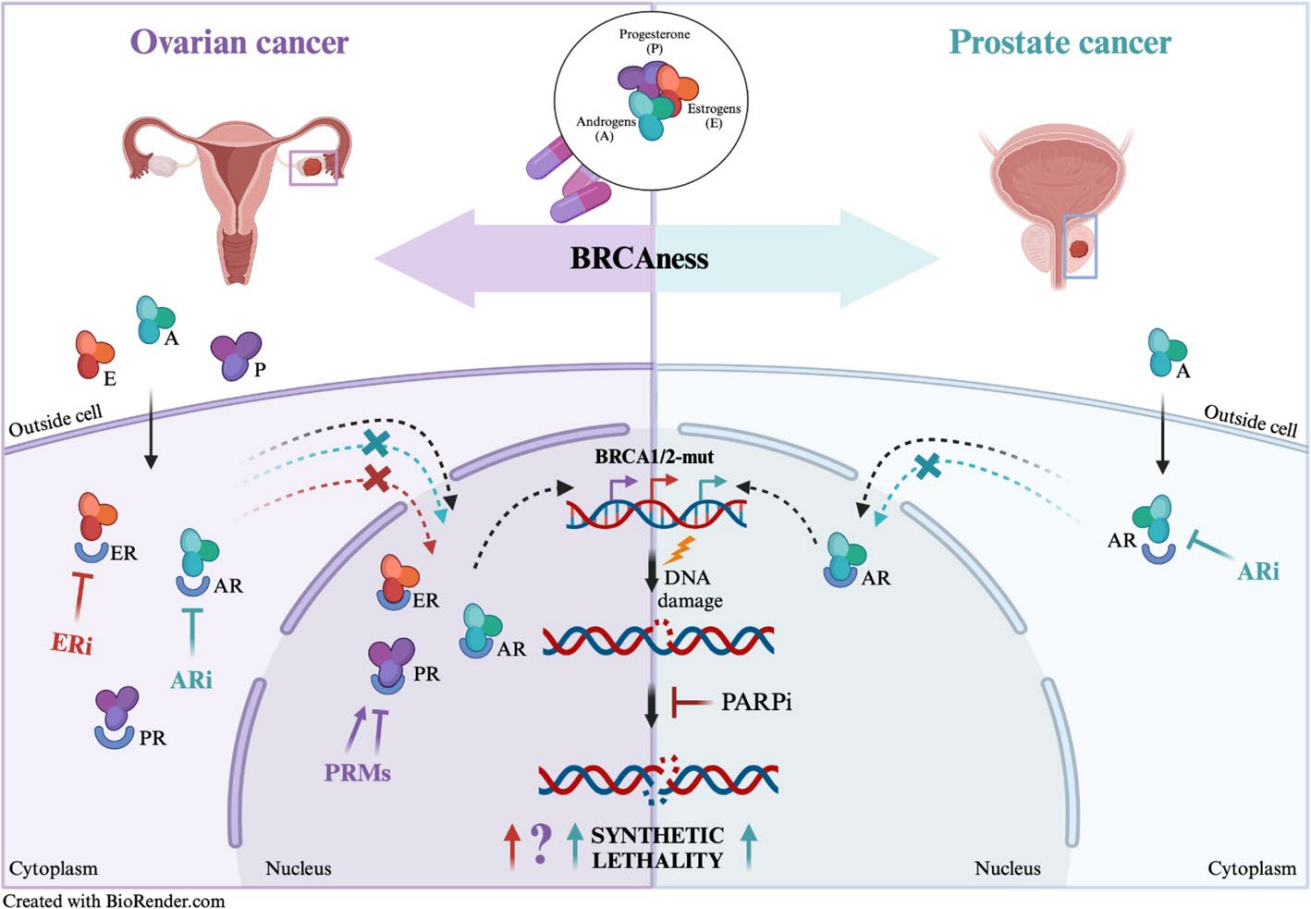
Interplay between hormones and DNA Repair in *BRCA*-deficient cancers. The figure indicates the intersection of hormone therapy with the concept of 'BRCAness' in the context of ovarian (left side) and prostate (right side) cancer. In the nucleus, the DNA carrying *BRCA1/2* mutations undergoes damage that can’t be repaired by the homologous recombination-based system. The inhibition of PARP, a key enzyme in the repair of single-strand DNA breaks, leads to synthetic lethality in these mutated cells, resulting in cell death. Modulation of estrogen (E), androgen (A) and progesterone (P) can influence the therapeutic landscape. Once the hormones enter inside the cells, they bind to their respective receptor (R) and might interact with different pathways and translocate to the nucleus to activate transcription of targeted genes. Inhibition of AR and ER blocks receptor translocation and might exert synthetic lethality with DNA damage response inhibitors, while the effect promoted by PR regulation through PR modulators (PRMs) remains still unclear

DNA damage in urogenital cancers is often pervasive, resulting from both endogenous metabolic processes and exogenous factors like radiation or chemotherapy [112]. Internally, DNA damage may arise from errors in DNA replication, reactive oxygen species (ROS) generated during cellular metabolism and natural cellular processes like hormone metabolism, particularly relevant in urogenital cancers [113, 114]. While the previous paragraph addressed errors in DNA replication, we now aim to delve into hormonal regulation and its implication for DNA damage and repair in OC and PC, two hormone-regulated malignancies (Fig. 1).

Hormonal regulation plays a significant role in the pathophysiology of OC. Ovarian hormones, primarily estrogen and progesterone, have been shown to affect cell proliferation, apoptosis, and DNA repair mechanisms [115]. A list of the main hormonal therapy and the respective clinical trials is presented below (Table 3).

**Table 3:**
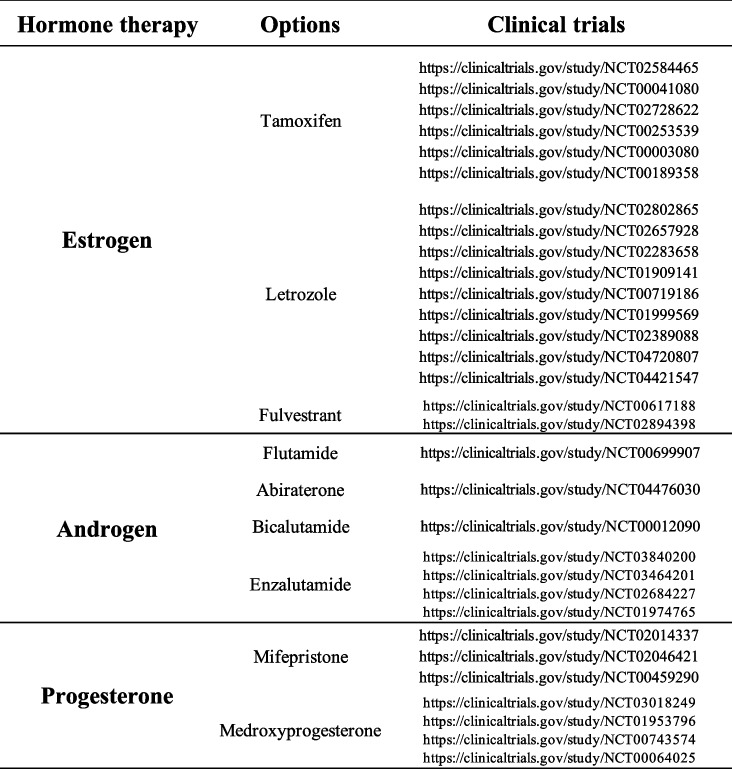
Hormonal therapy clinical trials in ovarian cancer. This table summarize the main hormonal treatment for ovarian cancer. Clinical trials available on the website: https://clinicaltrials.gov/ are listed for each main treatment option

Estrogen receptors (ER), primarily ERα and ERβ, are nuclear hormone receptors that mediate the effects of estrogen in target tissues. ERα is commonly associated with proliferative responses, while ERβ is thought to counteract these effects and is often linked with protective roles in cancer [116]. The mechanism of action of ERs involves the binding of estrogen, which facilitates their dimerization and subsequent binding to estrogen response elements (EREs) in the DNA. This binding initiates transcriptional regulation of various genes involved in cell growth, survival and differentiation [116]. In OC, the expression and activity of these receptors can significantly influence tumor behavior and patient prognosis. Recent studies, have highlighted the complex role of ERs in OC, demonstrating how ERα and ERβ can differentially regulate gene expression and contribute to cancer progression [117–119]. The link between ERs and DNA damage and repair mechanisms is an area of growing interest. Estrogen, through ER-mediated signaling, can influence the expression and activity of genes involved in DNA repair pathways, including HR and NHEJ [120, 121]. While a considerable amount of literature has explored the relationship between hormonal regulation and DNA repair pathways, only a few studies have delved deeply into this area [122]. Some of them have shown that estrogen-induced ER activation can modulate the expression of key DNA repair proteins, such as *BRCA1* and *RAD51*; this modulation can affect the efficiency of DNA repair mechanisms, influencing the sensitivity of OC cells to DNA-damaging agents [123]. Moreover, estrogen itself can be a source of DNA damage. Its metabolism can generate ROS and genotoxic metabolites, leading to DNA adducts and mutations and further implicating ER signaling in genomic instability [124]. Dysregulation of ERs, either through overexpression, mutation, or altered signaling pathways, can have significant implications in cancers, including OC [69]. Overexpression of ERα has been associated with increased tumor proliferation and poor prognosis. Conversely, loss or reduced expression of ERβ is often observed in OC and is thought to contribute to tumor aggressiveness and resistance to therapy [125]. Some researchers also highlighted the impact of ER dysregulation on the efficacy of hormonal therapies in *BRCA* mutant cancers showing that alterations in ER expression or function could lead to resistance to agents like selective estrogen receptor modulators (SERMs) and aromatase inhibitors (AIs) [126, 127]. On the other hand, progesterone has been shown to exert a protective effect against the development of OC. Progesterone receptors (PR), existing in two main isoforms PR-A and PR-B, are expressed in ovarian tissue and influence various cellular processes. While PR-B is typically associated with progesterone's classical reproductive actions, PR-A can act as a dominant negative inhibitor of both PR-B and ERs [128]. Progesterone receptors, which exist in two isoforms, upon binding progesterone, undergo conformational changes, dimerize, and translocate to the nucleus where they bind to progesterone response elements (PREs) in the DNA. This binding initiates the transcription of various genes involved in cell proliferation, differentiation and survival. The mechanism is tightly regulated and is subject to modulation by various co-factors and cellular contexts [129]. These mechanisms have been explored in different studies in which it was demonstrate that PR signaling can influence tumor behavior and response to therapy [129]. Currently, different clinical trials are focusing on PR signaling, especially evaluating the therapeutic potential of progesterone receptor modulators (PRMs), a new class of synthetic compounds, such as mifepristone (NCT02014337, NCT02046421). PRMs compete in the binding sites of the PR and can act both as agonist or antagonists respectively by inducing or inhibiting transcriptional activation of the PR making them more clinically relevant [130]. Of note, interest in studying the relationship between PR signaling and DNA damage and repair mechanisms is increasingly emerging. Progesterone has been shown to impact the expression of genes involved in DNA repair pathways, potentially influencing genomic stability, but the mechanism remains still unknown [131]. Some work suggests that progesterone-activated PRs may modulate the expression of key DNA repair proteins and influence the cellular response to DNA damage [132]. This modulation may have critical implications in the context of OC, where DNA repair capacity can significantly affect tumor behavior and treatment response. Dysregulation of PR signaling, either through altered receptor expression, mutations, or changes in ligand availability, can significantly affect OC since the overexpression or constitutive activation of PRs can lead to abnormal stimulation of target genes, contributing to tumorigenesis and progression [133]. Conversely, loss of PR expression or function has been associated with a more aggressive tumor phenotype and poorer prognosis in OC [132]. In OC, also AR can play a critical role despite its pivotal role in other malignancies such as PC. In the work by Chung and colleagues, the researchers point out that AR can contribute to tumorigenesis, metastasis and chemoresistance [134]. Although OC is more traditionally associated with estrogen and progesterone receptors, different other studies have highlighted AR involvement in OC. AR expression has been observed in various subtypes of OC and its activation has been linked to tumor growth and poor prognosis suggesting that targeting AR signaling, especially with AR antagonists such as enzalutamide, might represent a potential therapeutic strategy for OC [134–136]. In this context, abiraterone, a potent inhibitor of the enzyme CYP17A1, plays a crucial role in androgen biosynthesis and has been explored as a therapeutic agent in AR-driven cancers. The CORAL (Cancer of the OvaRy Abiraterone triaL) study (NCT04476030) was designed to evaluate the clinical activity of abiraterone in epithelial OC and it is the only one currently available in the literature. In this trial a subset of patients derived sustained clinical benefit providing important information regarding the role of AR-mediated signaling inhibition in patients with recurrent, advanced epithelial OC (EOC) [137]. This trial represents a significant effort to target the AR pathway in OC, potentially offering a new therapeutic avenue for patients with AR-positive tumors.

The intricate relationship between hormonal influences and DNA repair processes in OC offers insights into novel therapeutic strategies, including the use of hormonal therapies for which many clinical trials exist. These therapies aim to modulate or block hormonal effects, particularly those of estrogen [132]. SERMs, AIs and Gonadotropin hormone-releasing hormone (GnRH) analogs are among the primary classes of hormonal therapies used [138]. SERMs, such as tamoxifen, function by competitively binding to estrogen receptors, thereby inhibiting estrogen-mediated signaling in cancer cells. In different clinical trials were evaluated the effect of different hormones; for example tamoxifen showing promising results in patients with resistant OC (NCT02728622). Aromatase inhibitors, including drugs like letrozole and anastrozole work by inhibiting the aromatase enzyme responsible for estrogen synthesis. Even in this case some trials assess the effectiveness of letrozole in advanced OC resistant or not to platinum therapy (NCT04720807, NCT04421547), demonstrating its potential. Finally, GnRH analogues, used primarily in premenopausal women, suppress ovarian function, thus reducing estrogen production [139].

Despite the potential of hormonal therapies, several challenges exist in their clinical application. Recent clinical trials have been instrumental in advancing our understanding of hormonal therapies in OC. As describe above, there are different clinical trials already focusing on SERMs or aromatase inhibitors, but fewer on the use of hormonal therapy in combination with other treatments, such as PARPi or other targeted therapies aiming to enhance efficacy and overcome resistance. As demonstrated in the work by Hao and colleagues, the intricate interplay between non-classical estrogen signaling and HRR deficiency in OC underscores the pivotal role of ERα in this process. In this study they provide evidence that ERα can exert a repressive effect on HRR activity identifying HR as an ERα target, thereby leading to an increased chemosensitivity of OC cells. [140] This work highlights the potential benefits of hormone replacement therapy in ameliorating the outcomes of OC treatment which can maybe be enhanced by combinatorial treatment with DDRi. Targeting the effects of estrogen and progesterone offers several advantages in the treatment of OC. One of the primary advantages of hormonal therapy is its targeted approach as we described before, since it allows targeting the hormonal key players in the proliferation and survival of OC cells. Compared to traditional chemotherapy, hormonal therapies generally present a more favourable toxicity profile. They are associated with fewer and less severe side effects, making them a more tolerable treatment option for many patients. Finally, hormonal therapies have also shown particular efficacy in certain subtypes of OC, such as estrogen receptor-positive (ER +) or low-grade serous carcinomas [141]. Despite these advantages, one of the major challenges with hormonal therapy is the development of resistance. Over time, OC cells can adapt to these therapies, altering their receptor expression or activating alternative signaling pathways, but there are only a few review articles in which this type of resistance is investigated and no research works are available [118, 142]. Moreover, hormonal therapies are not universally effective across all OC subtypes. For example, high-grade serous OC (HGSOC), the most common and aggressive subtype, often does not effectively respond to hormonal therapy [118]. In summary, hormonal therapy in OC offers a targeted, less toxic alternative to traditional chemotherapy, with particular efficacy in certain cancer subtypes. However, challenges such as resistance development, limited efficacy in certain subtypes, and side effects cannot be overlooked. Thus, ongoing clinical trials and preclinical research are essential in addressing these challenges, improving therapeutic outcomes, finding alternatives to hormone therapy resistance and advancing personalized medicine approaches in the treatment of OC.

In PC, the AR is a member of the steroid hormone receptor family. AR signaling plays a fundamental role in physiological prostate development and function as well as in male morphologic development and configuration of the central neurons system [143]. The AR gene, located on the X chromosome, encodes 110 kDa protein composed of conserved DNA-binding domain and androgen-binding domain and a less conserved N-terminal transactivation domain [144]. AR influences transcription of androgen responsive genes. Recent findings showed the role of AR in PC growth and progression. In PC, AR can regulate cell proliferation, apoptosis, migration, invasion and cell differentiation [145]. Some studies also showed prognostic value of AR determined by immunohistochemistry, but the results are inconsistent and need to be verified [146]. PC development is dependent on androgens and androgen deprivation therapy (ADT) introduces an important therapeutic opportunity. ADT such as long-acting GnRH agonists (goserelin, histrelin, leuprolide, and triptorelin) or GnRH antagonists (degarelix), second-generation nonsteroidal AR antagonists (enzalutamide, apalutamide, and darolutamide) and the androgen biosynthesis inhibitor abiraterone are the first line therapy for patients with metastatic disease [147]. A list of the main hormonal therapy and the respective clinical trials is presented below (Table 4).

**Table 4:**
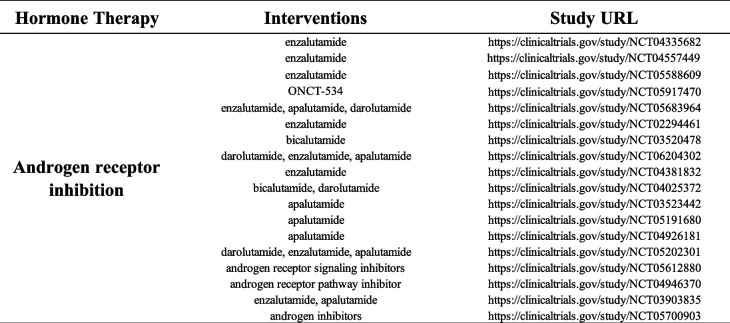
Hormonal therapy clinical trials in prostate cancer. This table summarize the main hormonal treatment for prostate cancer. Clinical trials available on the website: https://clinicaltrials.gov/ are listed for each main treatment option

In 1% of primary PC cases, mutations and amplifications of the AR are observed, with this rate increasing to approximately 60% in metastatic tumors [148]. These mutations predominantly occur in the androgen-building domain of AR, resulting in antiandrogens (e.g. bicalutamide, hydroxyflutamide, enzalutamide, and apalutamide) functioning as AR agonists. This enables cancer progression and contributes to PC resistance to androgen deprivation therapy. Cai and colleagues showed that the T878A mutation has been associated with resistance to abiraterone in a xenograft PC model [149]. Moreover, mutant AR has been identified in circulating cell-free DNA [150]. Splicing variants of AR have also been detected in PCs, with AR-V7 splice variant also detected at the protein level [151]. AR-V7 is frequently detected in CRPC (around 75% of cases) [152]. Armstrong and collaborators in the prospective multicentric study (The PROPHECY Study) showed that AR-V7 detected in the blood of mCRPC was associated with shorter PFS and OS after abiraterone or enzalutamide treatment [153] On the other hand, in circulating tumor cells (CTCs) from AR-V7-positive PC, taxanes are more effective, while in AR-V7-negative PC, the effect is comparable [154]. In recent years, there is emerging evidence that AR signaling and the DDR pathways are related. Goodwin and collaborators showed that DNA damage induces AR activity, and active AR induces cell survival after DNA damage, indicating reciprocal regulation between AR and DDR. The study also revealed the impact of AR on the expression of DNA repair genes, identifying DNA PKcs as a key target of AR after DNA damage [155]. Furthermore, combining ADT with radiotherapy has been standard care approach for PC. RNAseq and Chipseq analysis on the xenograft model of castration-resistant PC LNCaP-AR, treated by enzalutamide, revealed downregulation of DNA repair genes. Further analysis defined 32 direct targets for AR, including *RAD51C, MRE11A, CHEK1, LIG3*. AR signaling promotes double-strand DNA break repair and regulates the transcriptional program of DNA repair genes that promotes PC radio-resistance both in vitro and in vivo [156]. Previous studies showed that AR deprivation therapy enhances the effect of ionizing radiation by impairing NHEJ. However, AR signaling can also regulate HR genes. Asim and colleagues investigated the functional link between AR and HR, demonstrating decreased numbers of ionizing radiation-induced RAD51 foci in isogenic cells with low AR expression. Additionally, AR is required for effective ATM signaling mediated by MRE11. AR directly regulates HR activity, and androgen inhibition activates PARP signaling. Therefore, inhibition of AR is synthetically lethal with PARP inhibition in PC [157]. Furthermore, in PC, HR genes are frequently mutated, especially in mCRPC setting, offering potent therapeutic opportunities. The androgen inhibitor enzalutamide can suppress the expression of the HR genes, causing HR deficiency and BRCAness. This explains why enzalutamide and olaparib combination is effective in mCRPC patients and proves that also pharmaceutically induced BRCAness may expand the clinical use of PARPi [158]. A recent study showed that AR recruitment can be blocked by antineoplastic antibiotic mithramycin (MTM). MTM treatment caused the downregulation of AR target genes, including DDR genes. The study of Wang et al. discovered that MTM impaired DDR and enhanced effectiveness of the ionizing radiation and radiomimetic agent bleomycin [159]. Combining PARPi with AR inhibitors presents a powerful treatment option, as evidenced by several ongoing clinical studies. A phase 3 study is currently evaluating the PARPi niraparib in combination with apalutamide or abiraterone acetate plus prednisone in mCRPC [160]. Additionally, ongoing clinical studies are investigating combinations of enzalutamide with nanoparticle-based drugs [161] and I-131–1095 radiotherapy [162]. There is an increasing evidence about the role of progesterone and estrogen in the PC [163]. Recent findings indicated the potential oncogenic effects of progesterone in PC, with elevated progesterone levels associated with poor clinical outcomes in both castration-resistant and hormone-sensitive PC patients (HSPC). An increase in progesterone levels in the plasma of CRPC and HSPC patients was associated with poor clinical outcomes. Progesterone can activate canonical and non-canonical AR target genes, and inhibition of 3b-hydroxysteroid dehydrogenase 1 (3bHSD1) can suppress the oncogenic effects of progesterone [164]. Prostate tissues express both ERα and ERβ [165] and PC development depends also on estrogen signaling. Estrogen can increase the occurrence of androgen-induced PC [166]. Ricke and colleagues showed on a mice model that prostates from ERβ-knockout (βERKO) mice underwent carcinogenesis and the prostates from ER alpha-knockout mice remained free of disease [167]. Taking together ERβ is a tumor suppressor, and its inhibition leads to the prostate hyperplasia and tumor development. Therefore anti-estrogens and SERMS may reduce the risk of PC development in cases with high levels of ERβ [168]. ERα is also associated with the invasion and migration of PC cells [169]. Lombardi and colleagues demonstrated that PC3 cells express ERα and ERβ, with activation of ERβ influencing the expression of β-catenin and promoting proliferation of PC3 cells. Treatment with PKF 118–310, a drug that disrupts the β-catenin/TCF/LEF (T-cell-specific transcription factor/lymphoid enhancer-binding factor) complex, blocked the effect of ER-β [170].

### Preclinical models for studying DNA damage and repair triggered by chemo-, targeted- and hormonal- agents

**Fig. 2:**
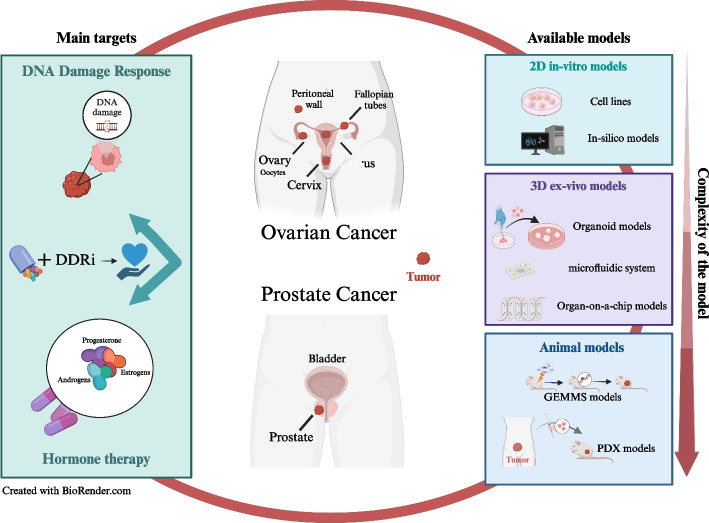
Innovative therapeutic strategies and models in ovarian and prostate cancer: from bench to bedside. The figure encapsulates the multifaceted approach to cancer research and treatment, specifically for ovarian and prostate cancer. On the left side, two primary therapeutic targets for these tumors are indicated: the DNA damage response (DDR) pathway, which can be inhibited by DDR inhibitors and hormone therapy, which involves the modulation of androgens, estrogens and progesterone levels. On the right side, the available research models for studying these targets are indicated basing on their complexity: on the top part 2D in vitro models, on the middle part more complex 3D ex vivo models, such as organoids, microfluidic systems, and organ-on-a-chip technologies, on the bottom part animal models including genetically engineered mouse (GEMMs) and patient-derived xenograft (PDX) models

Thus far, we have recognized the significance of investigating DNA damage and repair alongside hormonal regulation in urogenital cancers, particularly in tumors like OC and PC. To dig deeper into these mechanisms, comprehensive studies necessitate various preclinical models. These range from traditional methods like cell culture and animal models to computational simulations and ex vivo models. Additionally, advanced translational platforms such as organoids, microfluidics, and organ-on-a-chip systems are invaluable tools in elucidating these intricate processes (Fig. 2).

#### Investigating chemotherapy response using in vitro cell line studies

In vitro models, particularly cell line models, offer a simplified and controlled setting to study cancer biology, drug responses and genetic manipulations. We have extensively discussed how DDR pathways, particularly those involving HR and NHEJ, as well as hormonal regulation are often compromised especially in OC and PC. In this section we will delve into the main in-vitro models outlined in the literature, categorizing them based on the type of treatment and the development of resistance: chemotherapy, targeted therapy and hormone-based therapy both for ovary and prostate tumors.

#### In vitro chemotherapy-based studies

Despite advancements in research that introduce new therapeutic options, chemotherapy remains one of the primary treatments for OC and PC. Unfortunately, after the initial response, patients often develop resistance, highlighting the need for in vitro models to elucidate the mechanisms associated with these processes.

Cancer cell lines have been extensively utilized to investigate mechanisms of resistance to therapy, particularly in response to chemotherapy, which poses a significant challenge in treating OC and PC. To explore potential novel therapeutic strategies to overcome resistance, researchers have developed several cellular models with acquired resistance. By continuous exposure of cancer cell lines to the drug, researchers can observe the emergence of resistance and possibly identify the molecular changes that occur [49, 171, 172]. Consequently, several studies have focused on understanding the effects of chemotherapy alone or in combination with other treatments to elucidate the underlying mechanisms [74]. For instance, Bicaku and colleagues analyzed the response to carboplatin, cisplatin, and paclitaxel in OC survival. They treated 36 OC cell lines with these drugs, quantified IC50 levels and performed pre-treatment gene expression analyses correlating it with the IC50 levels biological pathway analysis. Results showed that cell line sensitivity to carboplatin, cisplatin, paclitaxel and their combination was associated with the expression of 77, 68, 64, and 25 biological pathways, respectively. From these results the study identified the Transcription/CREB pathway as one to be noted and that was associated with OC overall survival and cell line platinum sensitivity [74]. Similarly, Blanc-Durand and colleagues developed an assay to study HR in a chemotherapy treatment context. Their study found that HR deficiency, identified through a *RAD51* functional assay, was associated with higher response rates to neoadjuvant platinum chemotherapy and longer progression-free survival in OC [173]. Another study by Acland et al. aimed to identify molecular features specific to chemoresistance in OC using carboplatin-resistant OVCAR5 and CaOV3 cell line models. The results of this study revealed enhanced migratory and invasive potential in the chemoresistant lines compared to the parental ones. Moreover, through mass spectrometry analysis they found distinct metabolic and signaling perturbations in chemoresistant lines, including dysregulation in cytokine and type 1 interferon signaling. This shared feature between cell lines and patient-derived primary cells indicates a common molecular aspect of chemoresistance, providing insights for future research on molecular mechanisms of chemoresistance and related phenotypes [46].

In PC, cell lines with acquired resistance to taxanes were developed by cultivation with increasing concentration of the drug [174]. Lima and colleagues identified multiple mechanisms associated with docetaxel resistance such as ABCB1, an ATP-binding cassette transporter overexpression, moreover increased expression of the genes associated with androgen signaling, cell survival, and overexpression of non-coding RNAs [175]. ABCB1 overexpression was also identified as a main player of cabazitaxel cross resistance with docetaxel [176]. Furthermore, DNA-PKc, a crucial component of the DDR, was found to promote taxane resistance in mCRPC [177]. According published evidence there are several mechanisms contributed in docetaxel resistance development as P-glycoprotein which was overexpressed in cell lines resistant to docetaxel (DU-145R and 22Rv1R). Inhibition of P-glycoprotein with elacridar (a P-glycoprotein inhibitor) reversed the presence of resistant phenotype [178]. Mumenthaler and colleagues used a pharmacological inhibitor targeting the Pim kinase (SGI-1776), to evaluate the effect of Pim kinase activity on PC cell survival and resistance. They exploited a paclitaxel-resistant 22Rv1 cell line, showing that inhibition of Pim kinase activity sensitized taxane chemoresistant cells to apoptosis, indicating its potential as a therapeutic target in overcoming docetaxel resistance [179].

#### In vitro targeted therapy-based studies

HR alterations are prevalent in both OC and PC, presenting potential and novel therapeutic targets for both diseases. However, to improve therapy response and advance personalized medicine, there is a critical need to develop accurate in vitro models.

For OC, A2780, OVCAR-3 and SKOV-3 cell lines are among the most frequently utilized to investigate the effects of targeted therapy, given their well-established profiles regarding *BRCA1/2* mutations and other DDR-related genes [180]. For instance, numerous studies have employed these OC cell lines to elucidate the role of PARPi and/or ATM/ATR kinases in DNA repair processes [181]. Biegala and colleagues sought to understand olaparib resistance in OC and enhance its efficacy by investigating the cellular mechanisms of resistance. A key finding of their work was the development of an olaparib-resistant OC cell line (PEO1-OR) from *BRCA2* mutated PEO1 cells. The study revealed that PEO1-OR cells acquired resistance through *BRCA2* secondary mutations, upregulating HR repair-promoting factors and PARP1. Additionally, olaparib-resistant cells exhibited reduced sensitivity to ATR/CHK1 inhibitors, suggesting that combination therapy might resensitize them to PARPi, offering a potential strategy to overcome acquired resistance to PARPi in OC [182]. In another study, Fleury and colleagues investigated the sensitivity of HGSOC cell lines to PARPi, specifically olaparib. While PARPi sensitivity is commonly linked to HR deficiency, this study reveals a more complex scenario by demonstrating that downregulation of genes in the NER and MMR pathways also increases PARPi response. The highest sensitivity was observed when HR deficiency was concurrent with downregulation of either NER or MMR pathways, proposing a novel model for predicting PARPi sensitivity in patients [183]​​.

In PC, LNCaP and C4-2B resistant to olaparib also exhibited resistance to other clinically relevant PARPi (rucaparib, niraparib, talazoparib). These olaparib-resistant cell lines accumulated DNA damage compared to parental cells, suggesting potential mechanisms underlying resistance [184]. On a base of current treatment strategies, it is clinically relevant to study cross resistance between current PC therapies i.e. (taxanes) and olaparib. There is increasing evidence that cells with acquired chemoresistance to docetaxel report cross-resistance to olaparib. DU-145 with acquired resistance to docetaxel showed ABCB1 overexpression-mediated cross-resistance to olaparib [184]. Schaaf and colleagues obtained similar results regarding cross-resistance between taxanes and olaparib; in addition, they show that cells resistant to docetaxel retain sensitivity to enzalutamide and vice versa [185].

#### In vitro hormone therapy-based studies

Given the significance of hormonal regulation in OC and PC, the following section will focus on the in vitro models that elucidate the mechanism of action, therapy response and chemoresistance of therapies targeted to hormonal regulation.

In OC, the majority of the studies is focused on estrogen-based therapy. For instance, Chao and colleagues investigated estrogen impact on OC cell growth and survival, focusing on alterations in cell-cycle regulatory proteins. They treated ovarian adenocarcinoma cell lines, OC-117-VGH (estrogen receptor-deficient) and OVCAR3 (estrogen receptor-positive), with different estrogen concentrations and observed differential effects on cell-cycle regulatory proteins. While there were no significant changes in cyclin D1 and E expression, p16/INK4a and p27/KIP1 expression was higher in OC-117-VGH than in OVCAR3. This suggests that estrogen-mediated inhibitory effects on OC might be mediated through different pathways in ER-positive and ER-negative cell lines [139]. Similarly, Li and colleagues explored estrogen role in EOC proliferation. They found that estrogen stimulation increased OC cell proliferation and invasion, with higher expression of transient receptor potential channel C3 (TRPC3) observed in OC tissue compared to normal tissue, suggesting TRPC3 as a potential therapeutic target [186]. In the study by Lima and colleagues, the impact of sex hormones on ADAMTS 1 and 4 expression in OC cells was evaluated. Progesterone was found to significantly increase ADAMTS protein and mRNA levels, particularly in ES-2 cells, with this effect reversed by the progesterone receptor antagonist RU486. This study concluded that progesterone, through its receptor, modulates ADAMTS 1 and 4 levels in OC cell lines, thereby influencing cancer features [187]. Additionally, Pedernera and colleagues assessed the effect of sexual steroids, including progesterone, on cell survival in primary cultures of ovarian carcinoma. From the analysis of samples from 35 patients with various subtypes of epithelial OC, they found a significant reduction in cell survival after progesterone treatment, particularly in endometrioid ovarian carcinoma. This effect was notably pronounced in cells positive for PR, suggesting a crucial role for progesterone and its receptor in reducing the progression of endometrioid ovarian carcinoma [188]. Furthermore, Limaye and colleagues evaluated the effectiveness of AR inhibition in managing HGSOC with recurrent cases. This study focused on a patient with HGSOC who experienced multiple relapses, but achieved excellent disease control through AR inhibition by using bicalutamide. The results of this study support the potential of targeting AR signaling in the treatment of OC, especially in patients with recurrent disease after initial treatments​​ [189].

Androgen deprivation therapies are crucial for inhibiting PC progression. It is known that enzalutamide treatment decreases the expression of HR associated genes. Therapeutical approach where enzalutamide is followed by the olaparib showed significantly increased PC cell apoptosis [158]. Long-term culture in the presence of enzalutamide generated four genetically distinct enzalutamide-resistant AR-positive and AR-pathway dependent PC cell lines (CWR-R1, LAPC-4, LNCaP, VCaP). The transcriptomic characterization revealed deregulation in AR-associated and non-associated genes e.g. *TMEFF2* (Transmembrane protein with EGF-like and two follistatin-like domains-2), β-catenin (*CTNNB1*) pathways, *MT2A* (Metallothionein 2A) [190]. Additionally, studies by Liu and colleagues and Xu and colleagues demonstrated cross-resistance between enzalutamide and abiraterone in enzalutamide-resistant cells, with AR-V7 splicing variant identified as responsible for resistance to abiraterone. Inhibition of AR-V7 by niclosamide and enhancement of enzalutamide treatment by a novel HSP70 allosteric inhibitor, JG98, showed potential therapeutic benefits [191, 192]. Moreover, enzalutamide resistant cells remain sensitive to olaparib [193], which provides interesting therapeutical option for therapy resistant patients. On the other hand, van Soest and colleagues published abiraterone and enzalutamide cross-resistance with taxanes. Notably, docetaxel and cabazitaxel inhibit AR translocation to the nucleus [194].

The role of DNA repair in enzalutamide treatment response was proved by study Zhang and colleagues. In this study they used CRISP/Cas9 knockout (GeCKO) library to identify the DNA-damaging agent idarubicin responsible to overcame abiraterone and enzalutamide resistance in PC in vitro. Idarubicin can fight enzalutamide and abiraterone resistance by inhibition of XPA expression [195]. In addition to in vitro models, also in silico models are employed in biological research. These computational models are based on algorithms and simulations to analyze biological data and predict outcomes starting from molecular simulations to whole-genome analyses. They are particularly useful to analyze large datasets, such as genomic sequences, and to identify patterns, mutations, gene expression changes, response to certain treatments and they can even support personalized medicine by predicting the most effective treatment strategies based on individual patients’ genetic profiles [196].

#### In vivo mouse models: from GEMMs to PDXs

Animal models serve as crucial systems for studying cancer mechanisms, with genetically engineered mouse models (GEMMs) and patient-derived xenografts (PDXs) offering significant insights into tumor growth, metastasis, and therapeutic responses in an in vivo context. In the first case, GEMMs, featuring specific mutations in DDR genes, provide insights into the role of these genes in cancer development and progression. In the context of OC, different reviews focused their attention on these models highlighting their advantages in managing specific gene mutations and consequently being helpful in understanding the efficacy of a treatment especially for targeted therapies potentially leading to better clinical outcomes [197–199]. For example, Shi and colleagues demonstrate that the inactivation of multiple genes like *PTEN, TRP53, and RB1* in the ovarian surface epithelium of mice led to the development of type I low-grade OC, further emphasizing the utility of GEMMs in modelling the disease and its progression [200].

When studying PC, there are numerous GEMMs based on different genomic alterations relevant for PC and expressing different stages of the disease progression (for reviews see [201] and [202]). Ding and colleagues reported GEMMs by targeting *PTEN* and *TP53* to develop model with metastatic PC and genomic instability [203]. Downregulation of *CHK1*, which correlates with *ERG* expression in *PTEN* ± mice model, promoted high-grade prostatic intraepithelial neoplasia into invasive carcinoma [204].

On the other hand, PDXs generated by engrafting fresh human tumor fragments into immunodeficient mice, reflect patients' tissue histological and genetic characteristics [205]. The success rate of establishing PDXs depends on mouse origin and cancer tissue type, with higher rates observed in advanced or metastatic tumors. Indeed, the growth of PDX from primary tumors is around 2–10% while for advanced or metastatic tumors it is around 25–30% [206]. These models have been widely used in different tumors, for example animal models were used to study the effect of *BRCA1/2* mutations in OC [207].

In OC research, PDX models are established by transplanting fresh patient tumor tissues into mice, often at orthotopic sites, to mimic the tumor's original environment and preserve its heterogeneity and genetic landscape [208]. PDXs have been instrumental in assessing the efficacy of PARPi in OC: Chen and colleagues in 2022 were able not only to replicate in PDX the results of clinical trials such as NOVA (NCT01847274), PRIMA (NCT02655016), and SOLO I, suggesting the utility of these models in mimicking clinical responses, but they predict also PARPi efficacy better than *BRCA* mutational status or platinum sensitivity. Key findings include high *KRAS* expression correlating with PARPi sensitivity, *AKT1* enrichment indicating resistance, and low CA125 levels as potential PARPi efficacy indicators [209]. Additionally, Serra and colleagues investigated WEE1 and ATR inhibitors' efficacy in overcoming PARPi resistance in breast and ovarian cancers. Using patient-derived xeno-implant models, the study found that WEE1i response was associated with replication stress markers like STK11/RB1 and phospho-RPA, while ATRi response was associated to *ATM* mutations. The results suggest that targeting the replication stress response, particularly by WEE1i, can be an effective strategy to overcome PARPi resistance, even in tumors without homologous recombination repair deficiency. This approach provides important results and is under active testing in clinical trials [210].

In PC, there are several fundamental collection of PDX models like the MURAL collection [211] and the MD Anderson Prostate Cancer Patient-derived Xenograft Series (MDA PCa PDX) [212]. PDX models have demonstrated the antitumor activity of cabazitaxel in docetaxel- and enzalutamide-resistant tumors [177]. Therapy resistance is one of the biggest obstacles in PC therapy. Karkampouna et al. proposed a novel therapeutic strategy using multikinase inhibitors as ponatinib, sunitinib and sorafenib to overcome resistance to main PC therapies based on an androgen-dependent PCa PDX model [213].

Thus, differently from cell line models, PDX offers a platform for personalized medicine able also to recapitulate tumor heterogenity, crucial in studying the varied responses of different tumor cells to DNA damage and the efficacy of repair mechanisms. As far as PDXs present more advantages compare to cell lines, they also present several limitations: the establishment of PDX models is time-consuming, costly and resource-intensive since the growth rate of human tumors in mice can be slow, and not all patient samples successfully engraft and it requires specialized facilities [214]. Moreover, while PDX models maintain many aspects of the original tumor microenvironment, the immune component is significantly altered due to the immunodeficient nature of the host mice and this limitation can affect the study of immunological aspects of DDR. In addition, the use of animals in research brings ethical considerations and requires strict adherence to regulatory guidelines. Finally, while PDXs are valuable for preclinical studies, translating findings from these models to clinical outcomes can be challenging.

While animal models (syngeneic models) are widely used for the study of PARPi or other targeted therapies, we acknowledge that only few studies testing ICI in OC and PC are available, and even less works if considering possible combinatorial treatment with other drugs. Grabosch and colleagues assessed in vivo the response to anti-PD-L1 antibody and cisplatin either as single agents or in combination in EOC. The present study revealed that anti-PD-L1 targeted immunotherapy, when administered alone, exhibits remarkable efficacy against most aggressive models, even if this effect is tumor-dependent. It is important to note that cisplatin alone has the ability to modulate the immune microenvironment. Nevertheless, the combination of cisplatin with immune therapy appeared as the key for increasing mice survival rates in models of aggressive tumors and recurrent disease [215]. Also in the more recent work by Meng and colleagues, syngeneic mouse models were used to evaluate the therapeutic response of anti-PD-L1 therapy in OC, confirming how the effect of immunotherapy alone is limited, while the possible combination with PARPi such as niraparib, can improve the outcome [216]. Similarly to OC, for PC only few studies can be found [217]. Czernin and colleagues studied the synergistic effect of ^225^Ac-PSMA617 and anti-PD-1 antibody on a model of C57BL/6-mice bearing syngeneic RM1-PGLS tumors. The results of the study demonstrate synergic antitumor effect of PSMA RNT plus PD-1 blockade [217]. Eximond and colleagues tested also the triple combination anti-CTLA-4 + anti-PD-1 + RT in the model of syngeneic CRPC mouse. Their study showed that two ICIs in combination with RT had a stronger effect in comparison with monotherapy [218]. In general ICI therapy has only a moderate effect in PC. But there is an evidence that ADT might sensitize tumors to the checkpoint blockade by enhancing CD8 T cell function in mice model. Study on mouse implanted with PD-1 resistant tumors showed that enzalutamide is able to sensitize these mice to anti-PD-L1 antibody therapy by direct effect of androgen deprivation on immune cells in the tumor [219].

Overall these works suggest that combinatorial strategies for ICI, including both chemotherapy or targeted therapies, should be taken into considerations both for OC and PC to increase ICI effect.

#### Patient-derived 3D models: organoids, microfluidics and organ-on-a-chip

While previous models have contributed significantly to our understanding of ovarian and PCs, they fall short in fully capturing the complexity of human tumor microenvironment. To bridge this gap, translational models like organoids, microfluidics, and organ-on-a-chip systems have emerged as pivotal tools in cancer research. These models represent a significant milestone, particularly microfluidics and organ-on-a-chip systems, which integrate living human cells within a micro-engineered environment, simulating the physiology and mechanics of human organs. In details, microfluidics involves the manipulation of fluids at a microscale in channels with dimensions of tens to hundreds of micrometres, allowing precise control of the cellular microenvironment and facilitating the study of cellular responses under various physiological conditions [220]. Organ-on-a-chip systems, an extension of microfluidic technology, integrate cell cultures in a micro-engineered environment to mimic the structure and function of human organs. These systems can replicate key aspects of an organ’s microarchitecture and biomechanical properties, providing a more physiologically relevant model for studying disease processes [221]. The use of the microfluidic models has been instrumental in studying OCs, replicating tumor microenvironment and providing insights into tumor invasion and drug testing [222]. Despite their advantages, these systems are not without limitations. First, the design and fabrication of microfluidic and organ-on-a-chip systems can be complex and costly; they are optimized for small-scale experiments and the translation to clinical applications is challenging and not immediate. Finally, these systems often involve intricate techniques and precise control of experimental conditions [223].

OC and PC research has been hampered by the lack of suitable in vitro model systems. The most noteworthy translational model is the organoid one, as a self-organizing three-dimensional cell cultures generated from isolated pluripotent stem cells or progenitor cells of a patient’s tumor or non-tumor tissue [224]. Organoids closely mimic the architecture, functionality and genetic landscape of the original tissue, bridging the gap between traditional in vitro models and in vivo studies, becoming an indispensable tool in both basic research and clinical applications [225]. The genesis of organoid technology is largely attributed to the pioneering work of Hans Clevers, who has opened new avenues in studying a wide array of organs. Clevers and his team first demonstrated the potential of organoids in modeling the gut, showing that a single Lgr5 + stem cell from the adult mouse intestine could grow into a self-organizing structure that recapitulates the intestinal epithelium in vitro [226]. This revolutionary work illuminated the path for organoid research across various organ systems including the brain, gut, liver, prostate and ovaries [227], providing moreover new models for drug testing and for understanding disease mechanisms at a cellular level.

Focusing in particular on OC, due to the high degree of heterogeneity, organoid establishment and maintenance in culture was not easy. In this context, the literature is plenty of studies focusing on their establishment and different protocols were published and are still improving [228–230]. Moreover, there are also many works in which these organoids provide a means to investigate the unique tumor microenvironment of OC, including the study of tumor initiation, progression, metastasis and drug resistance [231]. One of the first relevant study in this field is the one from Kopper and colleagues in which OC organoids have been used to model different subtypes of the disease, including HGSOC being thus crucial in studying subtype-specific characteristics and responses to treatment [232]. The primary objective of their research was to establish a diverse panel of OC organoids that accurately reflect the various subtypes of OC, including HGSOC, which is the most common and aggressive form of the disease. These organoids were developed from tumor samples of patients with different OC subtypes, ensuring that the models encompassed a wide range of genetic and histological variations seen in actual patient tumors. A critical aspect of their study was the successful maintenance of the histopathological and genetic characteristics of the original tumors in the organoids demonstrating that they retained key features of OC, including specific genetic mutations, gene expression profiles, and histological structures, making them highly representative of the in vivo condition. The second main point of this work is that ovarian organoids were also employed to evaluate responses to various chemotherapeutic agents and targeted therapies showing that organoids' responses to these treatments mirrored clinical outcomes, demonstrating their potential as predictive models for personalized medicine. In other studies, organoids have been employed in high-throughput drug screening to identify potential therapeutics for OC. Nanki and colleagues developed expandable OC organoids and, after demonstrating their ability to model various subtypes of OC and to reflect the heterogeneity of the disease, employed them for drug sensitivity and resistance testing [233]. Of note, in this work they successful developed organoids in less than 3 weeks, capturing the characteristics of different histological cancer subtypes and replicating the primary tumors' mutational landscape. Furthermore, one organoid with a *BRCA1* pathogenic variant, showed higher sensitivity olaparib and platinum drugs and an organoid derived from clear cell OC exhibited resistance to conventional drugs, including platinum drugs, paclitaxel, and olaparib [233]. The potential of organoids in the evaluation of the molecular mechanism underlying OC was also demonstrated in the work from Wang and collaborators, where RNA sequencing of cisplatin-resistant and -sensitive OC organoids revealed higher *FBN1* expression in resistant samples. From further investigations they found that *FBN1*'s is involved in energy stress, angiogenesis, and chemoresistance and thanks to these results, they were able to identify the FBN1/VEGFR2/STAT2 signaling axis as a key mediator in these processes, suggesting potential therapeutic strategies targeting *FBN1* combined with antiangiogenic drugs for OC treatment [234]. Overall, these works suggest that organoids can accurately mirror the biology of the tumor of origin and can be exploited for high-throughput drug screening, identifying potential therapeutics and elucidating drug resistance mechanisms [225, 232].

It is well known that cells with stem-like potential represent a potential source to create patient-derived organoids (PDOs); in the case of PC, mainly basal cells, that show high proliferation and self-renewal and CD133 and CD44 phenotype compared to luminal cells, contribute to organoid establishment [235]. In the first PDO models, only basal cells reconstitute a prostate gland. In 2014 Karthaus and colleagues described the development of an R-spondin1-based culture method. This method admits a long-term propagation of murine and human prostate epithelium consisting of fully differentiated CK5 + basal and CK8 + luminal cells [236]. These protocols allowed cultured PDOs from prostate tissues, but they did not recapitulate AR signaling, which is essential for prostate development and also for PC progression and therapy. By adding Epidermal Growth Factor (EGF), Noggin, and R-spondin 1 to the growth medium, Drost and colleagues were able to generate long term growing organoids that functionally recapitulate AR signaling [237].

In general, successful generation of PDOs cultures struggles with many pitfalls. Organoid cultures from PC biopsies have variable growth rates caused probably by the high heterogeneity of the disease [238]. PC PDOs also show low efficiency in their establishment (15–20%) [239]. Servant and collaborators generated organoids from 81 PC patient samples. The success rate was around 44% for tissues from metastatic prostatectomy and around 28% for tissues from transurethral resection of the prostate [240]. In the study of Puca and colleagues, organoids from metastatic tissue of 25 PC patients were generated with a success rate of only 16% and the organoids were classified as neuroendocrine PC [241]. There is a strong evidence that PC organoids grow at different rates depending on the tissue of origin and clinico-pathological features of the patients’ tissue [240]. Because of their slow growth and low success rate, there is a need to optimize the protocol for generation of PDOs. Gao and colleagues established in 2014 for the first time the long term fully characterized cultured PC organoid platform derived from advanced and metastatic PC tissues, which recapitulates molecular diversity of PC and showed *TMPRSS2-ERG* fusion, *SPOP* mutation, *SPINK1* overexpression, and *CHD1* loss as well as mutations in DNA repair pathway. These PDOs showed common features for advanced PC such as *TP53* and *RB* loss, AR signaling, while mirroring the tumor of origin both at the genetic and phenotypic levels [242].

In conclusion, the versatility of organoids extends beyond disease modeling to regenerative medicine. Organoids offer a promising avenue for tissue regeneration and personalized medicine, including the potential for organ transplantation and the development of patient-specific treatment plans. Their ability to mimic patient-specific disease phenotypes makes them ideal for precision medicine applications, revolutionizing our ability to model human diseases and test therapeutic interventions with unprecedented precision and relevance.

## Conclusions and perspectives

In this review, we emphasize the significance of genomic instability, DNA damage and repair mechanisms, synthetic lethality, and hormonal regulation in OC and PC as well as the importance to use precise in vivo and in vitro models to study these signaling pathways. Understanding these factors is essential for improving diagnosis, treatment and outcomes in patients with urogenital cancers. One key aspect we delves into is the hormonal regulation and its implication for urogenital cancers treatment and resistance especially in the context of DNA damage and repair due to its significant impact on both the development and progression of these hormonal-related diseases. Since hormones such as estrogen, progesterone and androgens play important roles in urogenital function and pathology, their dysregulation can lead to enhanced proliferation of cancer cells and contribute to carcinogenesis. Thus, it is imperative to understand the interplay between hormonal pathways and DNA damage repair mechanisms in the context of OC and PC. Deciphering the role of hormones could facilitate the development of personalized medicine by identifying novel tailored treatments that might effectively circumvent the onset of resistance based on each patient's distinct cancer profile.

In this review we also discuss two of the main challenges for cancer therapy: therapy response and chemoresistance development, both involving DDR. Advances in understanding DDR mechanisms have led to the development of targeted therapies, such as PARPi, and to foster the design of novel therapeutic approaches to overcome acquired resistance. This focus on DNA damage and repair mechanisms is crucial for advancing research in precision medicine and understanding individual variations in DDR pathways in urogenital cancers could help adapting therapies to specific genetic profiles and optimizing therapeutic outcomes. In addition to this point, we have also emphasised the role of ICIs both in OC and PC, especially showing how their effect can be increased when in combination with other agents like ATR inhibitors, which could yield synergistic antitumor effects in patients with limited response to conventional therapies. Thus, further exploration and optimization of combination therapies could extend the benefits of ICIs to a broader patient population.

Lastly, we highlight the importance of utilizing advanced models to study these mechanisms, as they provide important insights into molecular pathways. Animal models and 3D ex vivo models have provided significant advancements in the field of OC and PC research. Starting from animal models, we highlighted how GEMMS and PDXs play pivotal roles in cancer research by providing in vivo systems that closely mirror human tumor biology allowing researchers to study the molecular and cellular mechanisms of cancer and following tumor progression and drug response. Transitioning to 3D technologies, we highlight microfluidics and organ-on-a-chip, which replicate structure and function while enabling fluid manipulation, providing a physiologically relevant model for disease processes. In this context, we wanted to shed light especially on the organoids, which have emerged as a critical bridge between in vitro and in vivo studies. Organoids can mimic the architecture and genetic landscape of the source tissue, enabling the study of disease mechanisms, drug responses and tumor evolution with a level of precision and relevance that was previously unattainable. In OC, organoids effectively tackle disease heterogeneity, modeling different subtypes, like HGSOC and facilitating studies on tumor characteristics and treatment responses. Diverse platforms of OC organoids that retain the genetic and histopathological features of the original tumors have been created, making them suitable for personalized medicine approaches, being used for drug sensitivity testing and elucidating molecular mechanisms underlying cancer. Also PC research has benefited from organoid technology, utilizing basal cells with stem-like potential to establish PC organoids, that mimic AR signaling—a key factor in PC progression. Despite the low establishment efficiency and variable growth rates, significant strides have been made in fully characterizing cultured prostate organoids. Thus, these systems offer detailed insights into tumor biology and are instrumental for therapeutic efficacy and toxicity assessments. In the quest for new cancer treatments, organoids serve as a powerful tool, allowing for more personalized therapy development and reducing reliance on animal testing, thereby expediting translation from bench to bedside. Nonetheless, challenges regarding complexity, cost, and scalability for clinical applications persist and are in constant development and improvement.

By integrating advanced technologies and more reliable models, we might advance our understanding on the interplay between response to DNA damage and hormonal regulation in urogenital cancers and develop more effective and personalized therapeutic options. Overall, the integration of multidisciplinary approaches will be essential for addressing the challenges posed by these complex diseases to improve patient care in the era of personalized medicine.

## Data Availability

Not applicable.

## Abbreviations

3bHSD1: 3b-hydroxysteroid Dehydrogenase 1
AI: Aromatase Inhibitor
ADTs: Androgen Deprivation Therapies
AR: Androgen Receptor
AR-V7: Androgen Receptor splice variant-7
BER: Base Excision Repair
CRPC: Castration Resistance Prostate cancer
CTCs: Circulating Tumor Cells
CTLA-4: Cytotoxic T-lymphocyte-associated protein 4
DDR: DNA Damage Response
DDRi: DNA Damage Response Inhibitor
DNA: Deoxyribonucleic Acid
DSBs: Double strand Breaks
EGF: Epidermal Growth Factor
ER: Estrogen Receptor
EREs: Estrogen Response Elements
GEMMs: Genetically engineered mouse models
GnRH: Gonadotropin hormone-Releasing Hormone
HGSOC: High-Grade Serous Ovarian Cancer
HSPC: Hormone Sensitive Prostate cancer
HR: Homologous Recombination
HRR: Homologous Recombination Repair
mCRPC: Metastatic Castration Resistant Prostate cancer
mPC: Metastatic Prostate cancer
MMR: Mismatch Repair
MRI: Magnetic Resonance Imaging
MTM: Mithramycin
NHEJ: Non-Homologous End Joining
NER: Nucleotide Excision Repair
OC: Ovarian cancer
PC: Prostate cancer
PARP: Poly-ADP Ribose Polymerase
PARPi: Poly-ADP Ribose Polymerase inhibitor
PCA3: Prostate cancer Antigen-3
PD-1: Programmed cell death protein 1
PD-L1: Programmed cell death-ligand 1
PDXs: Patient-derived xenografts
PDOs: Patient Derived Organoids
PET: Positron Emisssion Tomography
PHI: Prostate Health Index
PSA: Prostate Specif Antigen
PSMA: Prostate Specific Membrane Antigen
PR: Progesterone Receptor
PREs: Progesterone Response Elements
PRMs: Progesterone Receptor Modulators
ROS: Reactive Oxygen Species
SERMs: Selective Estrogen receptor modulators
SSBs: Single strand Breaks
TRPC3: Transient receptor potential channel C3

## References

[1] Holtedahl K (2023). Symptoms and signs of urogenital cancer in primary care. BMC Prim Care.

[2] Siegel RL (2023). Cancer statistics, 2023. CA Cancer J Clin.

[3] Helleday T, Eshtad S, Nik-Zainal S (2014). Mechanisms underlying mutational signatures in human cancers. Nat Rev Genet.

[4] Hanahan D (2022). Hallmarks of cancer: new dimensions. Cancer Discov.

[5] Lord CJ, Ashworth A (2016). BRCAness revisited. Nat Rev Cancer.

[6] González-Martín A (2019). Niraparib in patients with newly diagnosed advanced ovarian cancer. N Engl J Med.

[7] Mateo J (2019). A decade of clinical development of PARP inhibitors in perspective. Ann Oncol.

[8] Hanahan D, Weinberg RA (2011). Hallmarks of cancer: the next generation. Cell.

[9] Ali AT, Al-Ani O, Al-Ani F (2023). Epidemiology and risk factors for ovarian cancer. Prz Menopauzalny.

[10] Gaona-Luviano P, Medina-Gaona LA, Magaña-Pérez K (2020). Epidemiology of ovarian cancer. Chin Clin Oncol.

[11] Hunn J, Rodriguez GC (2012). Ovarian cancer: etiology, risk factors, and epidemiology. Clin Obstet Gynecol.

[12] Walker M, Jacobson M, Sobel M (2019). Management of ovarian cancer risk in women with. CMAJ.

[13] Budiana ING, Angelina M, Pemayun TGA (2019). Ovarian cancer: Pathogenesis and current recommendations for prophylactic surgery. J Turk Ger Gynecol Assoc.

[14] Liberto JM (2022). Current and emerging methods for ovarian cancer screening and diagnostics: a comprehensive review. Cancers (Basel).

[15] Matsas A (2023). Tumor markers and their diagnostic significance in ovarian cancer. Life (Basel).

[16] Dochez V (2019). Biomarkers and algorithms for diagnosis of ovarian cancer: CA125, HE4, RMI and ROMA, a review. J Ovarian Res.

[17] Dochez V (2019). Efficacy of HE4, CA125, risk of malignancy index and risk of ovarian malignancy index to detect ovarian cancer in women with presumed benign ovarian tumours: a prospective, multicentre trial. J Clin Med.

[18] Mathieu KB (2018). Screening for ovarian cancer: imaging challenges and opportunities for improvement. Ultrasound Obstet Gynecol.

[19] Ledermann JA (2018). First-line treatment of ovarian cancer: questions and controversies to address. Ther Adv Med Oncol.

[20] Raja FA, Chopra N, Ledermann JA (2012). Optimal first-line treatment in ovarian cancer. Ann Oncol.

[21] Menon U, Karpinskyj C, Gentry-Maharaj A (2018). Ovarian cancer prevention and screening. Obstet Gynecol.

[22] Chien J, Poole EM (2017). Ovarian cancer prevention, screening, and early detection: report from the 11th biennial ovarian cancer research symposium. Int J Gynecol Cancer.

[23] Guo T (2021). Cellular mechanism of gene mutations and potential therapeutic targets in ovarian cancer. Cancer Manag Res.

[24] Maioru OV (2023). Developments in genetics: better management of ovarian cancer patients. Int J Mol Sci.

[25] Ramus SJ, Gayther SA (2009). The contribution of BRCA1 and BRCA2 to ovarian cancer. Mol Oncol.

[26] Gorodetska I, Kozeretska I, Dubrovska A (2019). Genes: the role in genome stability, cancer stemness and therapy resistance. J Cancer.

[27] Stewart MD (2022). Homologous recombination deficiency: concepts, definitions, and assays. Oncologist.

[28] Mangogna A (2023). Homologous recombination deficiency in ovarian cancer: from the biological rationale to current diagnostic approaches. J Pers Med.

[29] Lin Y (2022). Metabolic reprogramming of the tumor immune microenvironment in ovarian cancer: a novel orientation for immunotherapy. Front Immunol.

[30] Rajwa P (2023). Prostate cancer risk, screening and management in patients with germline BRCA1/2 mutations. Nat Rev Urol.

[31] Gandaglia G (2021). Epidemiology and prevention of prostate cancer. Eur Urol Oncol.

[32] Wang G (2018). Genetics and biology of prostate cancer. Genes Dev.

[33] David MK, Leslie SW. Prostate Specific Antigen. 2022 Nov 10. In: StatPearls [Internet]. Treasure Island: StatPearls Publishing; 2024. 32491427

[34] Lepor A, Catalona WJ, Loeb S (2016). The prostate health index: its utility in prostate cancer detection. Urol Clin North Am.

[35] Cui Y (2016). Evaluation of prostate cancer antigen 3 for detecting prostate cancer: a systematic review and meta-analysis. Sci Rep.

[36] Shah S (2021). BRCA mutations in prostate cancer: assessment, implications and treatment considerations. Int J Mol Sci.

[37] Hofman MS (2020). Prostate-specific membrane antigen PET-CT in patients with high-risk prostate cancer before curative-intent surgery or radiotherapy (proPSMA): a prospective, randomised, multicentre study. Lancet.

[38] Tolkach Y, Kristiansen G (2018). The heterogeneity of prostate cancer: a practical approach. Pathobiology.

[39] Mateo J (2015). DNA-repair defects and olaparib in metastatic prostate cancer. N Engl J Med.

[40] Rebello RJ (2021). Prostate cancer. Nat Rev Dis Primers.

[41] de Bono J (2020). Olaparib for metastatic castration-resistant prostate cancer. N Engl J Med.

[42] Boussios S (2022). BRCA mutations in ovarian and prostate cancer: bench to bedside. Cancers (Basel).

[43] Rajan A (2021). Deregulated estrogen receptor signaling and DNA damage response in breast tumorigenesis. Biochim Biophys Acta Rev Cancer.

[44] Prados-Carvajal R (2021). Preventing and overcoming resistance to PARP inhibitors: a focus on the clinical landscape. Cancers (Basel).

[45] Hanahan D, Weinberg RA (2000). The hallmarks of cancer. Cell.

[46] Acland M (2022). Chemoresistant cancer cell lines are characterized by migratory, amino acid metabolism, protein catabolism and IFN1 signalling perturbations. Cancers (Basel).

[47] Huang R, Zhou PK (2021). DNA damage repair: historical perspectives, mechanistic pathways and clinical translation for targeted cancer therapy. Signal Transduct Target Ther.

[48] Negrini S, Gorgoulis VG, Halazonetis TD (2010). Genomic instability–an evolving hallmark of cancer. Nat Rev Mol Cell Biol.

[49] Alatise KL, Gardner S, Alexander-Bryant A (2022). Mechanisms of drug resistance in ovarian cancer and associated gene targets. Cancers (Basel).

[50] Sharma P (2017). Primary, adaptive, and acquired resistance to cancer immunotherapy. Cell.

[51] Patch AM (2015). Whole-genome characterization of chemoresistant ovarian cancer. Nature.

[52] Imyanitov E, Sokolenko A (2021). Mechanisms of acquired resistance of BRCA1/2-driven tumors to platinum compounds and PARP inhibitors. World J Clin Oncol.

[53] Aldea M (2021). Overcoming resistance to tumor-targeted and immune-targeted therapies. Cancer Discov.

[54] Kondrashova O (2017). Secondary somatic mutations restoring. Cancer Discov.

[55] Hurley RM (2021). Characterization of a RAD51C-silenced high-grade serous ovarian cancer model during development of PARP inhibitor resistance. NAR Cancer.

[56] Xu J (2023). RAD51D secondary mutation-mediated resistance to PARP-inhibitor-based therapy in HGSOC. Int J Mol Sci.

[57] Bashashati A (2013). Distinct evolutionary trajectories of primary high-grade serous ovarian cancers revealed through spatial mutational profiling. J Pathol.

[58] Karimi F (2023). Liquid biopsy in ovarian cancer: advantages and limitations for prognosis and diagnosis. Med Oncol.

[59] Pereira E (2015). Personalized circulating tumor DNA biomarkers dynamically predict treatment response and survival in gynecologic cancers. PLoS ONE.

[60] Alahdal M (2023). Current advances of liquid biopsies in prostate cancer: Molecular biomarkers. Mol Ther Oncolytics.

[61] Patel M (2020). The role of poly(ADP-ribose) polymerase inhibitors in the treatment of cancer and methods to overcome resistance: a review. Cell Biosci.

[62] Abida W (2017). Prospective genomic profiling of prostate cancer across disease states reveals germline and somatic alterations that may affect clinical decision making. JCO Precis Oncol.

[63] Huang XZ (2020). Efficacy and prognostic factors for PARP inhibitors in patients with ovarian cancer. Front Oncol.

[64] Alameddine Z (2023). A Meta-analysis of randomized clinical trials assessing the efficacy of PARP inhibitors in metastatic castration-resistant prostate cancer. Curr Oncol.

[65] Chandrasekaran A, Elias KM (2021). Synthetic lethality in ovarian cancer. Mol Cancer Ther.

[66] Neiger HE, Siegler EL, Shi Y (2021). Breast cancer predisposition genes and synthetic lethality. Int J Mol Sci.

[67] Hopkins JL, Lan L, Zou L (2022). DNA repair defects in cancer and therapeutic opportunities. Genes Dev.

[68] Alenezi WM (2023). Genetic analyses of DNA repair pathway associated genes implicate new candidate cancer predisposing genes in ancestrally defined ovarian cancer cases. Front Oncol.

[69] Omoike OE (2021). A cross-sectional study of the association between perfluorinated chemical exposure and cancers related to deregulation of estrogen receptors. Environ Res.

[70] Wu Y (2023). Clinical application of PARP inhibitors in ovarian cancer: from molecular mechanisms to the current status. J Ovarian Res.

[71] Miller RE, El-Shakankery KH, Lee JY (2022). PARP inhibitors in ovarian cancer: overcoming resistance with combination strategies. J Gynecol Oncol.

[72] Konstantinopoulos PA, Lheureux S, Moore KN (2020). PARP inhibitors for ovarian cancer: current indications, future combinations, and novel assets in development to target DNA damage repair. Am Soc Clin Oncol Educ Book.

[73] Alayev A (2016). Estrogen induces RAD51C expression and localization to sites of DNA damage. Cell Cycle.

[74] Bicaku E (2012). In vitro analysis of ovarian cancer response to cisplatin, carboplatin, and paclitaxel identifies common pathways that are also associated with overall patient survival. Br J Cancer.

[75] Foster KI (2023). Clinical implications of tumor-based next-generation sequencing in high-grade epithelial ovarian cancer. Cancer.

[76] Abida W (2019). Genomic correlates of clinical outcome in advanced prostate cancer. Proc Natl Acad Sci U S A.

[77] Lukashchuk N (2023). Impact of DNA damage repair alterations on prostate cancer progression and metastasis. Front Oncol.

[78] CrestaMorgado P, Mateo J (2022). Clinical implications of homologous recombination repair mutations in prostate cancer. Prostate.

[79] Zhang W (2020). Role of the DNA damage response in prostate cancer formation, progression and treatment. Prostate Cancer Prostatic Dis.

[80] Mateo J (2020). Genomics of lethal prostate cancer at diagnosis and castration resistance. J Clin Invest.

[81] Robinson D (2015). Integrative clinical genomics of advanced prostate cancer. Cell.

[82] Pritchard CC (2016). Inherited DNA-repair gene mutations in men with metastatic prostate cancer. N Engl J Med.

[83] Risdon EN (2021). PARP inhibitors and prostate cancer: to infinity and beyond BRCA. Oncologist.

[84] Vasquez JL (2020). Inhibition of base excision repair by natamycin suppresses prostate cancer cell proliferation. Biochimie.

[85] Kaufman B (2015). Olaparib monotherapy in patients with advanced cancer and a germline BRCA1/2 mutation. J Clin Oncol.

[86] Mateo J (2020). Olaparib in patients with metastatic castration-resistant prostate cancer with DNA repair gene aberrations (TOPARP-B): a multicentre, open-label, randomised, phase 2 trial. Lancet Oncol.

[87] Abida W (2020). Non-BRCA DNA damage repair gene alterations and response to the PARP inhibitor rucaparib in metastatic castration-resistant prostate cancer: analysis from the phase II TRITON2 study. Clin Cancer Res.

[88] Taylor AK (2023). PARP inhibitors in metastatic prostate cancer. Front Oncol.

[89] Drápela S (2020). The CHK1 inhibitor MU380 significantly increases the sensitivity of human docetaxel-resistant prostate cancer cells to gemcitabine through the induction of mitotic catastrophe. Mol Oncol.

[90] Tang Z (2021). ATR inhibition induces CDK1-SPOP signaling and enhances Anti-PD-L1 cytotoxicity in prostate cancer. Clin Cancer Res.

[91] Pardoll DM (2012). The blockade of immune checkpoints in cancer immunotherapy. Nat Rev Cancer.

[92] Tan S, Li D, Zhu X (2020). Cancer immunotherapy: pros, cons and beyond. Biomed Pharmacother.

[93] Zhang H (2021). Regulatory mechanisms of immune checkpoints PD-L1 and CTLA-4 in cancer. J Exp Clin Cancer Res.

[94] Pandey P (2022). Revolutionization in cancer therapeutics via targeting major immune checkpoints PD-1, PD-L1 and CTLA-4. Pharmaceuticals (Basel).

[95] Wojtukiewicz MZ (2021). Inhibitors of immune checkpoints-PD-1, PD-L1, CTLA-4-new opportunities for cancer patients and a new challenge for internists and general practitioners. Cancer Metastasis Rev.

[96] Indini A (2021). Immune-checkpoint inhibitors in platinum-resistant ovarian cancer. Cancers (Basel).

[97] Bogani G (2020). Immunotherapy for platinum-resistant ovarian cancer. Gynecol Oncol.

[98] Turinetto M (2021). The role of PARP inhibitors in the ovarian cancer microenvironment: moving forward from synthetic lethality. Front Oncol.

[99] Konstantinopoulos PA (2019). Single-arm phases 1 and 2 trial of niraparib in combination with pembrolizumab in patients with recurrent platinum-resistant ovarian carcinoma. JAMA Oncol.

[100] Drew Y (2024). Olaparib plus Durvalumab, with or without Bevacizumab, as Treatment in PARP Inhibitor-Naïve Platinum-Sensitive Relapsed Ovarian Cancer: A Phase II Multi-Cohort Study. Clin Cancer Res.

[101] Lee JM (2017). Safety and clinical activity of the programmed death-ligand 1 inhibitor durvalumab in combination with poly (ADP-Ribose) polymerase inhibitor olaparib or vascular endothelial growth factor receptor 1–3 inhibitor cediranib in women's cancers: a dose-escalation phase I study. J Clin Oncol.

[102] Zhu J, Yan L, Wang Q (2021). Efficacy of PD-1/PD-L1 inhibitors in ovarian cancer: a single-arm meta-analysis. J Ovarian Res.

[103] Sena LA (2021). Tumor frameshift mutation proportion predicts response to immunotherapy in mismatch repair-deficient prostate cancer. Oncologist.

[104] Hansen AR (2018). Pembrolizumab for advanced prostate adenocarcinoma: findings of the KEYNOTE-028 study. Ann Oncol.

[105] Antonarakis ES (2023). Pembrolizumab plus olaparib for patients with previously treated and biomarker-unselected metastatic castration-resistant prostate cancer: the randomized, open-label, phase III KEYLYNK-010 trial. J Clin Oncol.

[106] Graff JN (2021). KEYNOTE-641: a Phase III study of pembrolizumab plus enzalutamide for metastatic castration-resistant prostate cancer. Future Oncol.

[107] Gratzke C, et al. KEYNOTE-991: pembrolizumab plus enzalutamide and androgen deprivation for metastatic hormone-sensitive prostate cancer. Future Oncol. 2023. 10.2217/fon-2022-0776 36705526

[108] Petrylak DP (2021). KEYNOTE-921: phase III study of pembrolizumab plus docetaxel for metastatic castration-resistant prostate cancer. Future Oncol.

[109] Antonarakis ES (2020). Pembrolizumab for treatment-refractory metastatic castration-resistant prostate cancer: multicohort, open-label phase II KEYNOTE-199 study. J Clin Oncol.

[110] Appleton KM (2021). PD-1/PD-L1 checkpoint inhibitors in combination with olaparib display antitumor activity in ovarian cancer patient-derived three-dimensional spheroid cultures. Cancer Immunol Immunother.

[111] Yu EY (2023). Pembrolizumab plus olaparib in patients with metastatic castration-resistant prostate cancer: long-term results from the phase 1b/2 KEYNOTE-365 cohort a study. Eur Urol.

[112] Moretton A, Loizou JI (2020). Interplay between cellular metabolism and the DNA damage response in cancer. Cancers (Basel).

[113] Ding DN (2021). Insights into the role of oxidative stress in ovarian cancer. Oxid Med Cell Longev.

[114] Cucchi D, Gibson A, Martin SA (2021). The emerging relationship between metabolism and DNA repair. Cell Cycle.

[115] Li H (2021). Hormone therapy for ovarian cancer: emphasis on mechanisms and applications (Review). Oncol Rep.

[116] Shen Z (2017). Correlation between estrogen receptor expression and prognosis in epithelial ovarian cancer: a meta-analysis. Oncotarget.

[117] Langdon SP (2020). Estrogen signaling and its potential as a target for therapy in ovarian cancer. Cancers (Basel).

[118] Simpkins F, Garcia-Soto A, Slingerland J (2013). New insights on the role of hormonal therapy in ovarian cancer. Steroids.

[119] Faratian D (2011). Trastuzumab and pertuzumab produce changes in morphology and estrogen receptor signaling in ovarian cancer xenografts revealing new treatment strategies. Clin Cancer Res.

[120] Song L (2020). Cell type-specific genotoxicity in estrogen-exposed ovarian and fallopian epithelium. BMC Cancer.

[121] Vernier M (2020). Estrogen-related receptors are targetable ROS sensors. Genes Dev.

[122] Borella F (2023). Hormone receptors and epithelial ovarian cancer: recent advances in biology and treatment options. Biomedicines.

[123] Thasni KA (2008). Estrogen-dependent cell signaling and apoptosis in BRCA1-blocked BG1 ovarian cancer cells in response to plumbagin and other chemotherapeutic agents. Ann Oncol.

[124] Maleki J (2015). 17β-Estradiol stimulates generation of reactive species oxygen and nitric oxide in ovarian adenocarcinoma cells (OVCAR 3). Iran J Cancer Prev.

[125] Bogush TA (2018). Estrogen receptors alpha and beta in ovarian cancer: expression level and prognosis. Dokl Biochem Biophys.

[126] Schuster EF (2023). Molecular profiling of aromatase inhibitor sensitive and resistant ER+HER2- postmenopausal breast cancers. Nat Commun.

[127] Patel HK, Bihani T (2018). Selective estrogen receptor modulators (SERMs) and selective estrogen receptor degraders (SERDs) in cancer treatment. Pharmacol Ther.

[128] Jacobsen BM, Horwitz KB (2012). Progesterone receptors, their isoforms and progesterone regulated transcription. Mol Cell Endocrinol.

[129] Mauro LJ (2021). Progesterone receptors promote quiescence and ovarian cancer cell phenotypes via DREAM in p53-mutant fallopian tube models. J Clin Endocrinol Metab.

[130] Islam MS (2020). Selective progesterone receptor modulators-mechanisms and therapeutic utility. Endocr Rev.

[131] Rangsrikitphoti P (2023). Sex steroid hormones and DNA repair regulation: Implications on cancer treatment responses. J Steroid Biochem Mol Biol.

[132] Kim O (2020). Targeting progesterone signaling prevents metastatic ovarian cancer. Proc Natl Acad Sci U S A.

[133] Pu H (2023). Regulation of progesterone receptor expression in endometriosis, endometrial cancer, and breast cancer by estrogen, polymorphisms, transcription factors, epigenetic alterations, and ubiquitin-proteasome system. J Steroid Biochem Mol Biol.

[134] Chung WM (2021). Androgen/androgen receptor signaling in ovarian cancer: molecular regulation and therapeutic potentials. Int J Mol Sci.

[135] Manning-Geist BL (2022). Phase II study of enzalutamide in androgen receptor positive, recurrent, high- and low-grade serous ovarian cancer. Gynecol Oncol.

[136] Calvillo-Robledo A (2021). Simultaneous expression of steroid sulfatase and androgen receptor reduced overall survival of patients with epithelial ovarian tumors. J Ovarian Res.

[137] Banerjee S (2020). Abiraterone in patients with recurrent epithelial ovarian cancer: principal results of the phase II Cancer of the Ovary Abiraterone (CORAL) trial (CRUK - A16037). Ther Adv Med Oncol.

[138] Gadducci A, Cosio S, Genazzani AR (2006). Old and new perspectives in the pharmacological treatment of advanced or recurrent endometrial cancer: Hormonal therapy, chemotherapy and molecularly targeted therapies. Crit Rev Oncol Hematol.

[139] Chao KC (2013). The role of estrogen in the survival of ovarian tumors–a study of the human ovarian adenocarcinoma cell lines OC-117-VGH and OVCAR3. J Chin Med Assoc.

[140] Hao D (2019). Non-classical estrogen signaling in ovarian cancer improves chemo-sensitivity and patients outcome. Theranostics.

[141] Mitra S (2022). Hormonal therapy for gynecological cancers: how far has science progressed toward clinical applications?. Cancers (Basel).

[142] Sarwar S (2022). Insights into the role of epigenetic factors determining the estrogen response in estrogen-positive ovarian cancer and prospects of combining epi-drugs with endocrine therapy. Front Genet.

[143] Gelmann EP (2002). Molecular biology of the androgen receptor. J Clin Oncol.

[144] Aurilio G (2020). Androgen receptor signaling pathway in prostate cancer: from genetics to clinical applications. Cells.

[145] Culig Z, Santer FR (2014). Androgen receptor signaling in prostate cancer. Cancer Metastasis Rev.

[146] Tamburrino L (2012). Androgen receptor (AR) expression in prostate cancer and progression of the tumor: lessons from cell lines, animal models and human specimens. Steroids.

[147] Desai K, McManus JM, Sharifi N (2021). Hormonal therapy for prostate cancer. Endocr Rev.

[148] Kumar A (2016). Substantial interindividual and limited intraindividual genomic diversity among tumors from men with metastatic prostate cancer. Nat Med.

[149] Cai C (2011). Intratumoral de novo steroid synthesis activates androgen receptor in castration-resistant prostate cancer and is upregulated by treatment with CYP17A1 inhibitors. Cancer Res.

[150] Lallous N (2016). Functional analysis of androgen receptor mutations that confer anti-androgen resistance identified in circulating cell-free DNA from prostate cancer patients. Genome Biol.

[151] Sowalsky AG (2022). Assessment of Androgen Receptor splice variant-7 as a biomarker of clinical response in castration-sensitive prostate cancer. Clin Cancer Res.

[152] Sharp A (2019). Androgen receptor splice variant-7 expression emerges with castration resistance in prostate cancer. J Clin Invest.

[153] Armstrong AJ (2019). Prospective multicenter validation of androgen receptor splice variant 7 and hormone therapy resistance in high-risk castration-resistant prostate cancer: the PROPHECY study. J Clin Oncol.

[154] Antonarakis ES (2015). Androgen receptor splice variant 7 and efficacy of taxane chemotherapy in patients with metastatic castration-resistant prostate cancer. JAMA Oncol.

[155] Goodwin JF (2013). A hormone-DNA repair circuit governs the response to genotoxic insult. Cancer Discov.

[156] Polkinghorn WR (2013). Androgen receptor signaling regulates DNA repair in prostate cancers. Cancer Discov.

[157] Asim M (2017). Synthetic lethality between androgen receptor signalling and the PARP pathway in prostate cancer. Nat Commun.

[158] Li L (2017). Androgen receptor inhibitor-induced "BRCAness" and PARP inhibition are synthetically lethal for castration-resistant prostate cancer. Sci Signal.

[159] Wang S (2020). Mithramycin suppresses DNA damage repair via targeting androgen receptor in prostate cancer. Cancer Lett.

[160] Saad F (2021). Niraparib with androgen receptor-axis-targeted therapy in patients with metastatic castration-resistant prostate cancer: safety and pharmacokinetic results from a phase 1b study (BEDIVERE). Cancer Chemother Pharmacol.

[161] Schmidt KT (2022). A single-arm phase II study combining NLG207, a nanoparticle camptothecin, with enzalutamide in advanced metastatic castration-resistant prostate cancer post-enzalutamide. Oncologist.

[162] Afshar-Oromieh A (2017). Repeated PSMA-targeting radioligand therapy of metastatic prostate cancer with. Eur J Nucl Med Mol Imaging.

[163] Liao W (2023). Trends in estrogen and progesterone receptors in prostate cancer: a bibliometric analysis. Front Oncol.

[164] Hou Z (2022). Inhibiting 3βHSD1 to eliminate the oncogenic effects of progesterone in prostate cancer. Cell Rep Med.

[165] Belluti S, Imbriano C, Casarini L (2023). nuclear estrogen receptors in prostate cancer: from genes to function. Cancers (Basel).

[166] Bonkhoff H, Berges R (2009). The evolving role of oestrogens and their receptors in the development and progression of prostate cancer. Eur Urol.

[167] Ricke WA (2008). Prostatic hormonal carcinogenesis is mediated by in situ estrogen production and estrogen receptor alpha signaling. FASEB J.

[168] Steiner MS, Raghow S (2003). Antiestrogens and selective estrogen receptor modulators reduce prostate cancer risk. World J Urol.

[169] Kowalska K (2018). Estrogen receptor α is crucial in zearalenone-induced invasion and migration of prostate cancer cells. Toxins (Basel).

[170] Lombardi APG, Vicente CM, Porto CS (2020). Estrogen receptors promote migration, invasion and colony formation of the androgen-independent prostate cancer cells PC-3 through beta-catenin pathway. Front Endocrinol (Lausanne).

[171] Compadre AJ (2023). RAD51 Foci as a biomarker predictive of platinum chemotherapy response in ovarian cancer. Clin Cancer Res.

[172] Godwin AK (1992). High resistance to cisplatin in human ovarian cancer cell lines is associated with marked increase of glutathione synthesis. Proc Natl Acad Sci U S A.

[173] Blanc-Durand F (2023). A RAD51 functional assay as a candidate test for homologous recombination deficiency in ovarian cancer. Gynecol Oncol.

[174] Mohr L (2017). Generation of prostate cancer cell models of resistance to the anti-mitotic agent Docetaxel. J Vis Exp.

[175] Lima TS (2021). Molecular profiling of docetaxel-resistant prostate cancer cells identifies multiple mechanisms of therapeutic resistance. Cancers (Basel).

[176] Lombard AP (2017). ABCB1 mediates cabazitaxel-docetaxel cross-resistance in advanced prostate cancer. Mol Cancer Ther.

[177] Chao OS, Goodman OB (2021). DNA-PKc inhibition overcomes taxane resistance by promoting taxane-induced DNA damage in prostate cancer cells. Prostate.

[178] O'Neill AJ (2011). Characterisation and manipulation of docetaxel resistant prostate cancer cell lines. Mol Cancer.

[179] Mumenthaler SM (2009). Pharmacologic inhibition of Pim kinases alters prostate cancer cell growth and resensitizes chemoresistant cells to taxanes. Mol Cancer Ther.

[180] Xu J (2021). Arsenic compound sensitizes homologous recombination proficient ovarian cancer to PARP inhibitors. Cell Death Discov.

[181] Pillay N (2019). DNA replication vulnerabilities render ovarian cancer cells sensitive to poly(ADP-Ribose) glycohydrolase inhibitors. Cancer Cell.

[182] Biegała Ł (2021). PARP inhibitor resistance in ovarian cancer: Underlying mechanisms and therapeutic approaches targeting the ATR/CHK1 pathway. Biochim Biophys Acta Rev Cancer.

[183] Fleury H (2017). Cumulative defects in DNA repair pathways drive the PARP inhibitor response in high-grade serous epithelial ovarian cancer cell lines. Oncotarget.

[184] Lombard AP (2022). Olaparib-induced senescence is bypassed through G2-M checkpoint override in olaparib-resistant prostate cancer. Mol Cancer Ther.

[185] Schaaf ZA (2023). Therapeutic resistance models and treatment sequencing in advanced prostate cancer. Cancers (Basel).

[186] Li S (2020). Estrogen enhances the proliferation and migration of ovarian cancer cells by activating transient receptor potential channel C3. J Ovarian Res.

[187] Lima MA, da Silva SV, Freitas VM (2016). Progesterone acts via the progesterone receptor to induce adamts proteases in ovarian cancer cells. J Ovarian Res.

[188] Pedernera E (2019). Progesterone reduces cell survival in primary cultures of endometrioid ovarian cancer. J Ovarian Res.

[189] Limaye S (2020). A case report of androgen receptor inhibitor therapy in recurrent high-grade serous ovarian cancer. Oncotarget.

[190] Kregel S (2016). Acquired resistance to the second-generation androgen receptor antagonist enzalutamide in castration-resistant prostate cancer. Oncotarget.

[191] Liu C (2016). Niclosamide enhances abiraterone treatment via inhibition of androgen receptor variants in castration resistant prostate cancer. Oncotarget.

[192] Xu P (2023). Allosteric inhibition of HSP70 in collaboration with STUB1 augments enzalutamide efficacy in antiandrogen resistant prostate tumor and patient-derived models. Pharmacol Res.

[193] Handle F (2019). Drivers of AR indifferent anti-androgen resistance in prostate cancer cells. Sci Rep.

[194] van Soest RJ (2013). Cross-resistance between taxanes and new hormonal agents abiraterone and enzalutamide may affect drug sequence choices in metastatic castration-resistant prostate cancer. Eur J Cancer.

[195] Zhang Y (2022). Idarubicin combats abiraterone and enzalutamide resistance in prostate cells via targeting XPA protein. Cell Death Dis.

[196] Zhou Z (2021). The combination of cell cultured technology and in silico model to inform the drug development. Pharmaceutics.

[197] Zakarya R, Howell VM, Colvin EK (2020). Modelling epithelial ovarian cancer in mice: classical and emerging approaches. Int J Mol Sci.

[198] Tsang SI (2022). Experimental models for ovarian cancer research. Exp Cell Res.

[199] Hasan N, Ohman AW, Dinulescu DM (2015). The promise and challenge of ovarian cancer models. Transl Cancer Res.

[200] Shi M (2020). Inactivation of TRP53, PTEN, RB1, and/or CDH1 in the ovarian surface epithelium induces ovarian cancer transformation and metastasis. Biol Reprod.

[201] Adamiecki R (2022). In vivo models for prostate cancer research. Cancers (Basel).

[202] Arriaga JM, Abate-Shen C (2019). Genetically engineered mouse models of prostate cancer in the postgenomic era. Cold Spring Harb Perspect Med.

[203] Ding Z (2012). Telomerase reactivation following telomere dysfunction yields murine prostate tumors with bone metastases. Cell.

[204] Lunardi A (2015). Suppression of CHK1 by ETS family members promotes DNA damage response bypass and tumorigenesis. Cancer Discov.

[205] Pompili L (2016). Patient-derived xenografts: a relevant preclinical model for drug development. J Exp Clin Cancer Res.

[206] Slade D (2020). PARP and PARG inhibitors in cancer treatment. Genes Dev.

[207] Sun C (2020). MiR-509-3 augments the synthetic lethality of PARPi by regulating HR repair in PDX model of HGSOC. J Hematol Oncol.

[208] Hidalgo M (2014). Patient-derived xenograft models: an emerging platform for translational cancer research. Cancer Discov.

[209] Chen J (2022). Using Patient-Derived Xenograft (PDX) models as a 'Black Box' to identify more applicable Patients for ADP-Ribose Polymerase Inhibitor (PARPi) treatment in ovarian cancer: searching for novel molecular and clinical biomarkers and performing a prospective preclinical trial. Cancers (Basel).

[210] Serra V (2022). Identification of a molecularly-defined subset of breast and ovarian cancer models that respond to WEE1 or ATR inhibition, overcoming PARP inhibitor resistance. Clin Cancer Res.

[211] Risbridger GP (2021). The MURAL collection of prostate cancer patient-derived xenografts enables discovery through preclinical models of uro-oncology. Nat Commun.

[212] Palanisamy N (2020). The MD anderson prostate cancer patient-derived xenograft series (MDA PCa PDX) captures the molecular landscape of prostate cancer and facilitates marker-driven therapy development. Clin Cancer Res.

[213] Karkampouna S (2021). Patient-derived xenografts and organoids model therapy response in prostate cancer. Nat Commun.

[214] Jin J (2023). Challenges and prospects of patient-derived xenografts for cancer research. Cancers (Basel).

[215] Grabosch S (2019). Cisplatin-induced immune modulation in ovarian cancer mouse models with distinct inflammation profiles. Oncogene.

[216] Meng J (2021). Niraparib exhibits a synergistic anti-tumor effect with PD-L1 blockade by inducing an immune response in ovarian cancer. J Transl Med.

[217] Czernin J (2021). Immune-checkpoint blockade enhances 225Ac-PSMA617 efficacy in a mouse model of prostate cancerl. J Nucl Med.

[218] Eximond M, Wang J, Kirschner A (2023). Dual immune checkpoint therapy combined with radiotherapy treats castration-resistant prostate cancer. Int J Radiat Oncol Biol Phys.

[219] Guan X (2022). Androgen receptor activity in T cells limits checkpoint blockade efficacy. Nature.

[220] Halldorsson S (2015). Advantages and challenges of microfluidic cell culture in polydimethylsiloxane devices. Biosens Bioelectron.

[221] Danku AE (2022). Organ-on-a-chip: a survey of technical results and problems. Front Bioeng Biotechnol.

[222] Dadgar N (2020). A microfluidic platform for cultivating ovarian cancer spheroids and testing their responses to chemotherapies. Microsyst Nanoeng.

[223] Sood A (2023). Translational nanomedicines across human reproductive organs modeling on microfluidic chips: state-of-the-art and future prospects. ACS Biomater Sci Eng.

[224] Durinikova E, Buzo K, Arena S (2021). Preclinical models as patients' avatars for precision medicine in colorectal cancer: past and future challenges. J Exp Clin Cancer Res.

[225] Drost J, Clevers H (2018). Organoids in cancer research. Nat Rev Cancer.

[226] Clevers H (2016). Modeling development and disease with organoids. Cell.

[227] Sato T (2009). Single Lgr5 stem cells build crypt-villus structures in vitro without a mesenchymal niche. Nature.

[228] Maenhoudt N, Vankelecom H (2021). Protocol for establishing organoids from human ovarian cancer biopsies. STAR Protoc.

[229] Trillsch F (2023). Protocol to optimize the biobanking of ovarian cancer organoids by accommodating patient-specific differences in stemness potential. STAR Protoc.

[230] Graham O, et al. Generation and culturing of high-grade serous ovarian cancer patient-derived organoids*.* J Vis Exp. 2023;(191):10.3791/64878. 10.3791/64878 PMC10881225 36688549

[231] Raghavan S (2021). Microenvironment drives cell state, plasticity, and drug response in pancreatic cancer. Cell.

[232] Kopper O (2019). An organoid platform for ovarian cancer captures intra- and interpatient heterogeneity. Nat Med.

[233] Nanki Y (2020). Patient-derived ovarian cancer organoids capture the genomic profiles of primary tumours applicable for drug sensitivity and resistance testing. Sci Rep.

[234] Wang Z (2022). The Fibrillin-1/VEGFR2/STAT2 signaling axis promotes chemoresistance via modulating glycolysis and angiogenesis in ovarian cancer organoids and cells. Cancer Commun (Lond).

[235] Maitland NJ, Collins AT (2008). Prostate cancer stem cells: a new target for therapy. J Clin Oncol.

[236] Karthaus WR (2014). Identification of multipotent luminal progenitor cells in human prostate organoid cultures. Cell.

[237] Drost J (2016). Organoid culture systems for prostate epithelial and cancer tissue. Nat Protoc.

[238] Beshiri M (2023). Prostate organoids: emerging experimental tools for translational research. J Clin Invest.

[239] Horst EN (2021). Personalized models of heterogeneous 3D epithelial tumor microenvironments: ovarian cancer as a model. Acta Biomater.

[240] Servant R (2021). Prostate cancer patient-derived organoids: detailed outcome from a prospective cohort of 81 clinical specimens. J Pathol.

[241] Puca L (2018). Patient derived organoids to model rare prostate cancer phenotypes. Nat Commun.

[242] Gao D (2014). Organoid cultures derived from patients with advanced prostate cancer. Cell.

